# The Impact of Activin A on Fetal Gonocytes: Chronic Versus Acute Exposure Outcomes

**DOI:** 10.3389/fendo.2022.896747

**Published:** 2022-05-31

**Authors:** Sarah C. Moody, Penny A. F. Whiley, Patrick S. Western, Kate L. Loveland

**Affiliations:** ^1^ Centre for Reproductive Health, Hudson Institute of Medical Research, Clayton, VIC, Australia; ^2^ Department of Molecular and Translational Science, Monash University, Clayton, VIC, Australia

**Keywords:** activin A, signalling, fetal germ cell, fetal testis, differentiation

## Abstract

Activin A, a TGFβ superfamily member, is important for normal testis development through its actions on Sertoli cell development. Our analyses of altered activin A mouse models indicated gonocyte abnormalities, implicating activin A as a key determinant of early germline formation. Whether it acts directly or indirectly on germ cells is not understood. In humans, the fetal testis may be exposed to abnormally elevated activin A levels during preeclampsia, maternal infections, or following ingestion of certain medications. We hypothesized that this may impact fetal testis development and ultimately affect adult fertility. Germ cells from two mouse models of altered activin bioactivity were analysed. RNA-Seq of gonocytes purified from E13.5 and E15.5 *Inhba* KO mice (activin A subunit knockout) identified 46 and 44 differentially expressed genes (DEGs) respectively, and 45 in the E13.5 *Inha* KO (inhibin alpha subunit knockout; increased activin A) gonocytes. To discern direct effects of altered activin bioactivity on germline transcripts, isolated E13.5 gonocytes were cultured for 24h with activin A or with the activin/Nodal/TGFβ inhibitor, SB431542. Gonocytes responded directly to altered signalling, with activin A promoting a more differentiated transcript profile (increased differentiation markers *Dnmt3l, Nanos2* and *Piwil4*; decreased early germ cell markers *Kit* and *Tdgf1*), while SB431542 had a reciprocal effect (decreased *Nanos2* and *Piwil4*; increased *Kit*). To delineate direct and indirect effects of activin A exposure on gonocytes, whole testes were cultured 48h with activin A or SB431542 and collected for histological and transcript analyses, or EdU added at the end of culture to measure germ and Sertoli cell proliferation using flow cytometry. Activin increased, and SB431542 decreased, Sertoli cell proliferation. SB431542-exposure resulted in germ cells escaping mitotic arrest. Analysis of FACS-isolated gonocytes following whole testis culture showed SB431542 increased the early germ cell marker *Kit*, however there was a general reduction in the impact of altered activin A bioavailability in the normal somatic cell environment. This multifaceted approach identifies a capacity for activin A to directly influence fetal germ cell development, highlighting the potential for altered activin A levels *in utero* to increase the risk of testicular pathologies that arise from impaired germline maturation.

## Introduction

The complex processes governing the successful transformation of a primordial germ cell into a spermatogonial cell requires signals from the dynamic somatic milieu of the growing testis. There are gaps in our knowledge of these cues in the fetal testis which are particularly evident in the interval following assignment of a male fate and birth. In mouse, male sex determination initiates around embryonic day (E) 10.5, with *Sry* expression in pre-Sertoli cells which proliferate and surround the proliferating germ cells as testis cords are formed. Germ cells commit to the male fate by about E12.5 in response to signals from somatic cells (1, 2). From E13.5, these male germ cells, called gonocytes or pro-spermatogonia, enter mitotic arrest in an asynchronous manner to become uniformly quiescent by E15.5 (3). During this interval, transcripts that indicate their more differentiated status increase significantly, including *Nanos2*, *Dnmt3l* and *Piwil4*, while markers expressed in their less-differentiated precursors, such as *Kit*, *Nodal* and *Tdgf1*, decrease (4–6). These are hallmark indicators of the male germ cell genome transitioning to an epigenetically more stable state, as the piRNA pathway components, *Piwil4, Dnmt3l, Mov10l1, Tdrd1, Tdrd9*, are upregulated in a sex-specific manner.

**Figure 1:**
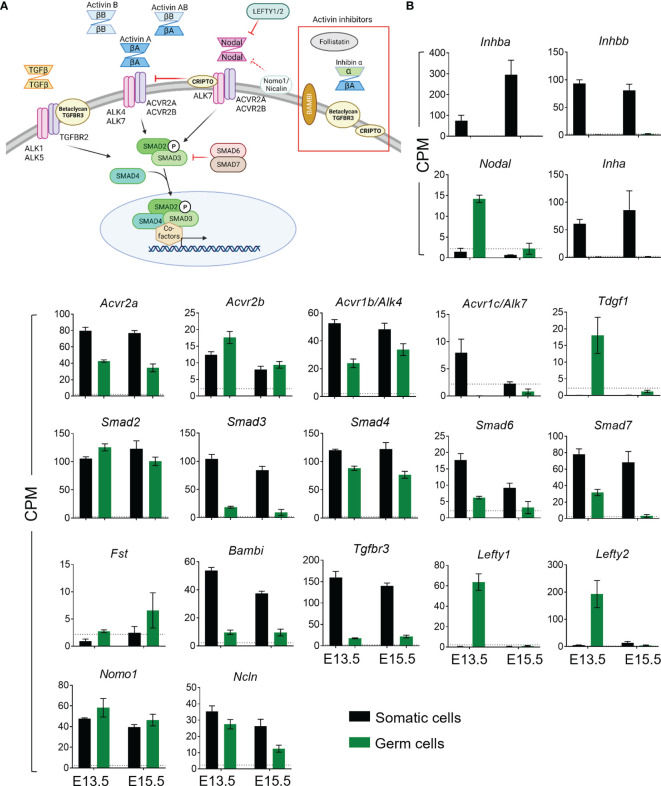
Activin and Nodal signalling component expression profiles in somatic and germ cells from wildtype E13.5 and E15.5 testes. **(A)** Overview of activin/Nodal/TGFβ signalling pathway and modulators. Dimeric ligands bind to two Type 2 receptors with a constitutively active serine-threonine kinase (STK) (purple) which then recruit, phosphorylate and thereby activate Type 1 receptor subunits with STK activity (pink). The complex can phosphorylate SMADs 2 and 3, and two of these complex with SMAD4 for transport into the nucleus, where interactions with nuclear co-factors effect changes in gene transcription. Crosstalk between family members is a feature of this pathway. This is mediated by the shared utilization of receptors (e.g. between activin A and Nodal), SMADs (common to activin/Nodal/TGFβ), and the inhibitory impact of Nodal pathway components (e.g. Cripto and Lefty1/2) on activin A signaling. **(B)** RNA-sequencing was performed on somatic and germ cell populations isolated from E13.5 and E15.5 *Inhba x Oct4-Gfp* mouse testes. The somatic cell data was published previously (9). Transcript levels of activin and Nodal ligands, type 1 and type 2 receptors, intracellular Smads, and activin and Nodal inhibitors in wildtype somatic (black columns) and germ cells (green columns) are shown in counts per million (cpm). Data are presented as mean ± SD. The detection limit for the *Inhba x Oct4-Gfp* RNA-Seq data set was 2.2 cpm (dotted line).

Proteins in the transforming growth factor β (TGFβ) superfamily produced by several testis cell types shape the growing fetal and postnatal testes and affect germ cell development. This superfamily contains over 30 different ligands, including transforming growth factor-betas (TGFβs), bone morphogenetic proteins (BMPs), activins, Nodal and growth and differentiation factors (GDFs) (7, 8). They share a conserved dimeric ligand structure, and signal through both shared and distinct signalling moieties, making the potential for signalling crosstalk and synergy of context-dependent importance (**Figure 1A**). For example, both activin A and TGFβ1 are implicated in stimulating germline exit from the cell cycle; genetically modified mice with decreased signalling by either one leads to a modest but significant increase in the proportion of germline cells that continue to proliferate at E15.5 (10, 11). However *in vitro* exposure to an inhibitor that blocks both pathways, SB431542, yielded a more robust outcome when testis fragments were cultured from E12.5 to E15.5 (12), suggesting that these pathways are partially redundant in the context of fetal germline maturation. In the context of human pregnancy, the premature elevation of activin A is an established indicator of pre-eclampsia that has been identified as early as the first trimester (13–15), the period of development in humans during which the germline initiates and progresses through sex-specific development. Understanding how disruptions to activin A signalling affect fetal germline development may provide clues to human reproductive pathologies.

In mouse and human, there are four activin subunits, inhibin βA (encoded by *Inhba)*, inhibin βB, inhibin βC and inhibin βE, which can form either hetero- or homo-dimeric ligands to signal. As with all other TGFβ superfamily members, the mature activin A protein consists of two INHBA subunits joined by a disulphide bond (16). Indicative of its importance, activin A mature protein subunits are 100% identical between these species. A mouse model with global knockout of the gene encoding the mature activin A subunit*, Inhba*, was first reported in 1995; pups with homozygous deletion of *Inhba* die within 24 hours of birth primarily due to their inability to suckle, amongst other defects that illustrate widespread contributions of activin A to fetal organ development (17). Within the mouse testis, *Inhba* transcript levels increase from E11.5 until shortly after birth (10). At postnatal day 0, *Inhba* KO mice have smaller testes, fewer Sertoli cells and higher gonocyte numbers compared with testes of wildtype littermates. This phenotype emerges after E13.5, with a small but significant increase in gonocyte numbers at E15.5 in KO testes (10), highlighting the potential for activin A to directly suppress germ cell proliferation.

Circumventing the neonatal lethality of *Inhba* KO mice, *Amhr2*-cre driven deletion of *Inhba* in Leydig cells resulted in smaller testes at E19.5, reduced coiling of the fetal testis cords and reduced Sertoli cell proliferation (18). This identified fetal Leydig cells as a key source of activin A. An *Sf1*-cre conditional knockout of *Inhba* in Sertoli and other somatic cells further revealed that activin A synthesized by gonocytes or immune cells did not rescue this phenotype (19). Adults with either of these conditional *Inhba* deletions had smaller testes, larger seminiferous tubule diameters, and tubules with abnormal or absent spermatogenesis. Such results demonstrated the potential for long term effects of reduced activin A on adult male fertility, some of which could be attributed to its roles in postnatal Sertoli cell proliferation and immunomodulatory functions (20–23).

More recently, activin A levels were shown to determine both steroidogenesis and lipid metabolism in the fetal testis. Activin A-deficient E13.5 and E15.5 testes in *Inhba* KO mice have drastically reduced levels of the *Hsd17b1* and *Hsd17b3* transcripts which encode the enzymes that convert androstenedione (A4) to testosterone. In the fetal testis, these enzymes are exclusively synthesized in Sertoli cells, and consequently, A4 produced from cholesterol in Leydig cells is not efficiently converted into testosterone (T) in *Inhba* KO testes. At E17.5 these testes exhibit an abnormal accumulation of lipid droplets within the testis cords and an elevated A4/T ratio (9), both indicating a profound impact of activin A signalling pathway on processes central to masculinization in a key developmental window (24).

The present study addresses the poorly understood question of how altered activin A signalling affects germ cell development, focussing on events that occur in the fetal testis after sex determination while testis *Inhba* transcript levels are rising and phenotypic changes in the *Inhba* KO testes are emerging between E13.5 and E15.5. Utilising a multi-pronged approach, we examined the impact of both the chronic (*in vivo*) and transient (*in vitro*) changes in activin A bioactivity on fetal male mouse germ cells. The results presented identify that both direct and indirect affects/mediators are likely to influence germline development depending on local levels of activin A during this key developmental window.

## Materials and Methods

### Animals

All animal procedures were carried out in accordance with the Australian Code of Practice for the Care and Use of Animals for Scientific Purposes under Monash University Animal Ethics Committee approval. Mice were housed at Monash Medical Centre Animal Facility under a 12-hour dark/light cycle and with food and water available *ad libitum*. For all experiments except RNA-Seq, timed-mates were set up between Swiss females and transgenic *Oct4-eGfp* males (OG2; pure 129T2svJ background) (25, 26). Females were checked daily and the presence of a vaginal plug marked as E0.5. At E13.5, pregnant females were culled by cervical dislocation and the uterine horns removed and placed in phosphate buffered saline (PBS). Embryos were removed and euthanised by decapitation and the developmental stage was determined by the time since mating, and fore- and hind-limb morphology. The gonad/mesonephros complex was dissected out of each embryo and the mesonephros removed. Sex of the embryo was identified by the absence or presence of testis cords in the gonads, visualised using an upright dissecting microscope (MZFLIII, Leica, Wetzlar, Germany).

For RNA-Seq, mice lacking the inhibin βA [encoded by *Inhba*; no activin A (17)] or inhibin alpha subunits [*Inha*; high activin A (27)] on a C57/Bl6 background were crossed with *Oct4-eGfp* mice (9, 25, 26). For each line, heterozygous timed mates were set up and fetal gonads from E13.5 and E15.5 embryos collected as above. Tails were collected from each embryo for commercial genotyping (Transnetyx, USA).

### Whole Gonad Culture

E13.5 testes were randomly assigned treatment groups. Testes were cultured on 0.4 µM Millicell cell culture inserts (Merck Millipore, Germany) in 6-well plates with each well containing 1.4 mL media (DMEM/F12, Thermo Fisher Scientific, Waltham, MA, USA; 10% FBS, Bovogen, Keilor East, VIC, Australia; 1% penicillin-streptomycin, Thermo Fisher Scientific) (28). Previous cell culture experiments with mouse postnatal day 6 and 15 Sertoli cells revealed SMADs localise to the nucleus following exposure to 5 ng/mL activin A (29). Further, the human germ cell-like line, TCam-2, is responsive to activin A at 5 ng/mL (30). However, the local concentration of activin A in the fetal testis is unknown, therefore we determined the optimum concentration of activin A by culturing whole testes with 0 (vehicle control; 4 µM HCl), 5, 25, 50 or 100 ng/mL human recombinant activin A (R&D Systems, Minneapolis, MN, USA) and activin-responsive genes measured. Following analysis of changes in activin A-responsive genes, testes were cultured in media containing 50 ng/mL activin A as the optimal dose (described in Results), or 10 µM SB431542 (Sigma-Aldrich, St Louis, MS, USA) and their respective controls (DMSO for SB431542). PBS was placed in the gaps between wells to maintain humidity. E13.5 testes were cultured for 48 hours with a full media change at 24 hours. Following culture, gonads were imaged using bright field and fluorescence using an Olympus IX70 inverted microscope to visualize gross gonad structure and GFP-positive germ cells. Gonads were removed from the membrane, washed in PBS and individually snap-frozen on dry ice for transcript analysis, fixed in 4% paraformaldehyde (PFA) for histological analysis, or dissociated for flow cytometry.

### Testis Dissociation and Germ Cell Isolation by Fluorescent Activated Cell Sorting (FACS)

For RNA-Seq experiments, paired testes from one embryo yielded a single biological replicate. From whole gonad cultures (E13.5 + 48h), single testes were a biological replicate. For E13.5 germ cell cultures, 6 - 10 paired testes were pooled. Testis dissociation and isolation of germ and somatic cells were performed as previously described (9). Briefly, testes were dissociated in 0.25% Trypsin-EDTA. Dissociation was halted with media containing 10% FBS. Cells were passed through a 35 µM strainer to obtain a single-cell suspension then centrifuged. Cell pellets were resuspended in 0.4% BSA/PBS and propidium iodide was added for exclusion of non-viable cells. GFP-positive and GFP-negative cells were sorted by Monash FlowCore staff using either an Influx or ARIA Fusion (BD Biosciences) machine. Sorted cells were pelleted, supernatant removed, and stored at -80°C for transcript analyses. Gonocyte cell culture is described below.

### EdU Incorporation and Flow Cytometry

This protocol was based on a previously published method using the Click-iT™ Edu Alexa Fluor™ kit (Thermo Fisher Scientific) (31). For EdU incorporation, a final concentration of 20 µM was added to culture media two hours before collection. Then testes were washed in PBS and dissociated in 0.25% Trypsin-EDTA at 37°C for 5 to 10 minutes. Dissociation was halted with DMEM/F12 containing 10% FCS, and the cells were passed through a 35 µm mesh cell strainer to obtain a single cell suspension. Following centrifugation and removal of supernatant, cells were resuspended in 4% PFA and fixed for 15 minutes at room temperature. After 3 washes in permwash (1X saponin-based permeabilisation reagent (Thermo Fisher Scientific) in 1% BSA/PBS) cells were stored in permwash for no more than one week before immunostaining. For all steps, solutions were made up in and washes done with permwash and performed at room temperature.

Cells were centrifuged and resuspended in 5% donkey serum (Sigma) for 15 minutes. Cells were incubated with anti-DDX4 (detection of germ cells; R&D Systems; AF2030; goat polyclonal; 1:100) and anti-DNMT3L (Abcam; ab194095; rabbit polyclonal; 1:200), or anti-SOX9 (Millipore; AB5535; rabbit polyclonal, 1:300) and anti-AMH (Anti-Mullerian Hormone; Santa Cruz; sc6886; 1:300), or anti-DDX4 and anti-SOX9 in combination for 45 minutes. Dissociated mesonephros was used as a negative control for DDX4 staining, and dissociated E13.5 ovaries were used as a negative control for SOX9 staining. Cells were washed twice then incubated 45 minutes with secondary antibodies (Donkey anti-rabbit biotin, Thermo Fisher Scientific; Donkey anti-goat Alexa Fluor 488, Thermo Fisher) diluted 1:300. Cells were washed twice then resuspended and incubated in the Click-iT reaction cocktail containing Alexa Fluor 647 for 30 minutes. Following two washes, samples were incubated with Streptavidin Pacific Blue (Thermo Fisher Scientific; 1:500) for 30 minutes. Cells were washed twice, then resuspended in 300 µL permwash containing 20 µg/mL propidium iodide (Sigma) to measure cellular DNA content. Samples were run on the same day on the BD Biosciences FACS CANTO-II at Monash FlowCore (Monash Health and Translational Precinct (MHTP) Node) and data analysed using FlowJo X.0.7 software (Ashland, OR, USA). Single, intact cells were analysed following gating using forward and side scatter characteristics, and DNA content.

### Double Immunofluorescence Staining of Mouse Testis Sections

Cultured and uncultured testes were fixed in 4% PFA for immunofluorescence (IF) analysis. After standard ethanol processing conditions, they were paraffin embedded and sectioned at 4 µM onto Superfrost Plus slides.

Unless stated, all steps were performed at room temperature. Sections on slides underwent dewaxing in histosol, followed by rehydration in a graded ethanol series (100%, 95% and 70% ethanol). Slides were briefly washed in water and incubated for 30 mins at 98°C in Citrate buffer (pH 6; DAKO) for antigen retrieval. After cooling, slides were rinsed in distilled water, washed once in PBS, then sections permeabilised in 0.1% Triton-X-100 (Merck) in PBS for 30 mins. Slides were washed twice in PBS and a wax circle drawn around sections using a PAP pen (Cederlane Laboratories, Burlington, Canada). Non-specific antibody binding was blocked by incubation with 10% donkey serum (Sigma-Aldrich) in 5% bovine serum albumin (BSA)/PBS for 1 hour. The blocking liquid was tapped off and primary antibodies in dual combination were diluted in 1% BSA/PBS and added to sections, with 1% BSA/PBS serving as a control lacking primary antibody. Primary antibodies against the following proteins were used: DNMT3L (Abcam; ab194094; 1:200), VASA (R&D Systems; AF2030; raised in goat; 1:400), VASA (Cell Signalling Technologies; 8761S; raised in rabbit; 1:400), cKIT (R&D Systems; AF1356; 1:100), Laminin (Sigma; L9393; 1:200) and AMH (Santa Cruz; sc6886; 1:200). Slides were incubated overnight at 4°C in a humid chamber. The next day, primary antibodies were removed, and slides washed 3 x 5 minutes in PBS. Secondary antibodies (Donkey anti-Rabbit Alexa Fluor 594, Invitrogen, A21207; Donkey anti-Goat Alexa Fluor 488, A11055) were diluted 1:300 in 1% BSA/PBS and added to sections for 2 hours. Slides were washed one time in 0.1% Triton-X-100 in PBS, then twice for 5 minutes each in PBS. DAPI (Thermo Fisher Scientific) was applied to sections at 5 µg/mL in PBS for 15 minutes. Following three washes in PBS, slides were mounted under glass coverslips with ProLong Gold Anti-fade Mountant (Thermo Fisher Scientific) and set overnight. Imaging was performed by Monash Histology Platform (MHTP node) using the VS120 Slide Scanner (Olympus, Tokyo, Japan) and images were processed using OlyVIA software (Olympus).

### Gonocyte Cell Culture

Following dissociation of Swiss x *Oct4-Gfp* E13.5 testes and isolation of gonocytes *via* FACS, germ cells were counted using a haemocytometer. In each well (48-well plate), 20,000 germ cells were added in 500 µL media (MEM-α, 10% FBS, 1% penicillin-streptomycin) containing 10 µM SB431542, 5 ng/mL activin A, or relevant vehicle control. A lower concentration of activin A was used compared with the whole testis cultures, as cells grown as a monolayer have been demonstrated to be robustly responsive to 5 ng/mL activin A (29, 30). Cells were cultured for 24 hrs in 5% CO_2_/air, after which the cells were collected for transcript analysis. Because the germ cells were lightly adherent, media and one PBS wash were collected to avoid losing cells, and 200 µL of 0.1% Trypsin-versene added per well and incubated for approximately 5 minutes or until all remaining cells were detached. Trypsin was quenched with media containing 10% serum, and all contents were transferred to a 1.5 mL tube containing the original media and PBS wash. Cells were centrifuged at 1020 g, supernatant removed, and cell pellets stored at -80°C.

### RNA Extraction, cDNA Synthesis and qRT-PCR

All RNA extractions and on-column DNase treatment were performed using the NucleoSpin RNA XS kit (Machery-Nagel, Germany) according to the manufacturer’s protocol. RNA concentration was quantified using a NanoPhotometer (Implen, Munchen, Germany). RNA was subjected to reverse transcription in a reaction containing 200 Units SuperScript III Reverse Transcriptase (Thermo Fisher Scientific), 50 ng random primers and 500 ng oligo dTs (Promega, Madison, USA) per sample. For whole gonads, 100 ng RNA was added to the reaction. For isolated cell cultures and cells isolated following culture, 40 ng and 15 ng respectively, was used in each cDNA reaction and RNaseOUT Recombinant Ribonuclease Inhibitor (Thermo Fisher Scientific) added to each 20 µL reaction as per the manufacturer’s protocol.

Real time PCR was conducted on the QuantStudio Fast Real-time PCR System at the MHTP Medical Genomics Facility (Clayton, Australia), and data generated using SDS software (Applied Biosystems). Each reaction contained power SYBR Green Master Mix (Thermo Fisher) and specific primer pairs (**Table 1**; Integrated DNA Technologies, Coralville, IA, USA) facilitating transcript measurements in 384 well plates. Primers pairs were designed to span exon-exon junctions or have pairs separated by an intron where possible. Each cDNA was diluted 1:20 or 1:10 for whole testes and isolated cells respectively. Every sample was measured in triplicate, and amplification of a single product was indicated by detection of a single peak in a melt curve analysis. Data were normalised to the *Canx* housekeeper gene (33) and analysed using the 2^-ΔCt^ method.

**Table 1:** Forward and reverse primers for qRT-PCR (SYBR Green).

Gene	Accession	Forward (5’- 3’)	Reverse (5’- 3’)
***Canx***	NM_001110499.1	TTCCAGACCCTGATGCAGA	TCCCATTCTCCGTCCATATC
***Piwil2***	NM_021308.2	TTGGCCTCAAGCTCCTAGAC	GAACATGGACACCAAACCTACA
***Piwil4***	NM_001368831.1	GGGGCTCGTTGTCCTTACCA	ACTGCCTTCATCAGGCGGAA
***Tdrd1***	NM_001002241.2	TCTTCCCACAGCACCATCTGTA	CACTCTTCACTTCAATGGCCT
***Tdrd9***	NM_029056.1	TGGCGAGTTGACCTTCCTGG	CTGAACGCCTCCACAAGTGC
***Dnmt3a***	NM_007872.4	GGCCCGTTACTTCTGGGGTA	TGGCTATTCTGCCGTGCTCC
***Dnmt3l***	NM_001284197.1	ATGATCAAGAGGGAGCGGGC	CGAGCCGTACACCAGGTCAA
***Mov10l1***	NM_031260.2	AAGAGTACCTGGTCATCGTCATCTC	CAGCAGTGCTTTGGGTCTTG
***Mvh***	NM_001145885.1	CATCGAATTGGACGCACTG	GGCAATCTCTTCTAGCCATGC
***Oct4***	NM_013633.3	GTTGGAGAAGGTGGAACCAA	CTCCTTCTGCAGGGCTTTC
***Kit***	NM_001122733.1	TCATCGAGTGTGATGGGAAA	GGTGACTTGTTTCAGGCACA
***Nodal***	NM_013611.5	ACATGTTGAGCCTCTACCGAGAC	AACGTGAAAGTCCAGTTCTGTCC
Tdgf1*	NM_011562.2	GGCCATTTCCAGTGCGTTT	GCAAGGTCTCTCCCAGCAAC
***Nanos2***	NM_194064.2	TCTCCATGGACCATTCACG	CTTCCTCTTATTCCTGATGGACA
***Sox9***	NM_011448.4	TGAACGCCTTCATGGTGTG	TTCTCGCTCTCGTTCAGCAG
***Mmp2***	NM_008610.3	TCGCTCAGATCCGTGGTGAG	TCATTCCCTGCGAAGAACACA
***Ccl17***	NM_011332.3	AATGTAGGCCGAGAGTGCTG	TGGCCTTCTTCACATGTTTG
***Cldn11***	NM_008770.3	AGTTCTCCCCTGCATCCGAA	TCACAGCACCGATCCAACCT
***Gja1***	NM_010288.3	AGGAGTTCCACCACTTTGGCG	AAATGAAGAGCACCGACAGCC
***Serpina5***	NM_172953.3	TCTTCACCACCCATGCTGAC	GAATGTGAAGATGGCTCCTGTG
***Hsd17b1***	NM_010475.2	CACTACCTGCGTGGTTATGAGC	GAAGCGGTTCGTGGAGAAGTAG

*Souquet et al., (32).

Additionally, transcript levels in isolated E13.5 germ cell cultures were measured using the Fluidigm Biomark™ 96x96 Dynamic Array IFC by the MHTP Medical Genomics Facility. Taqman assays (Thermo Fisher Scientific; **Table 2**) were used for amplification of specific transcripts. The geometric mean of two housekeeper genes, *Canx* and *Mapk* (33), was used for normalisation of data following a Pearson correlation between the two Ct values (R^2^>0.92). Data were normalised to the housekeepers, stable across samples, and analysed using the 2^-ΔCt^ method. Multiple experiments were analysed on the same array, accounting for the remaining samples and Taqman assays which make up the 96x96 array.

**Table 2:** Taqman assays for Fluidigm analysis.

Gene	Taqman assay
***Mapk***	Mm00442479_m1
***Canx***	Mm00500330_m1
***Nodal***	Mm00443040_m1
***Tdgf1***	Mm03024051_g1
***Lefty2***	Mm00774547_m1
***Dnmt3l***	Mm00457635_m1
***Slc43a3***	Mm00469627_m1
***Msi1***	Mm01203522_m1

### RNA-Sequencing

RNA-Sequencing was performed on gonocyte and somatic cells isolated from E13.5 and E15.5 *Inhba x Oct4-Gfp*, and germ cells isolated from E13.5 *Inha* x *Oct4-Gfp* wildtype and knockout testes. RNA sample quality was assessed on the Agilent 2100 Bioanalyzer using the Eukaryote total RNA Pico Kit, providing a measure of RNA integrity (RIN). All samples were high quality (RIN 8.4 – 9.9). Double stranded cDNA was prepared from 2-20 ng total RNA using Trio RNA-Seq or RNA-Seq V2 kits and SPIA amplification (Tecan/NuGEN, Leek, The Netherlands). These methods both use full length linear amplification to minimise bias. RNA-Seq libraries were then prepared with unique barcodes to allow multiplexing during sequencing. Illumina single end sequencing was performed on the HiSeq 3000 or NextSeq2000 (Illumina, San Diego, CA, USA). All RNA quality control, library preparation, and sequencing were performed by staff at the MHTP Medical Genomics Facility.

### RNA-Sequencing Analysis

Sequencing from E13.5 and E15.5 *Inhba x Oct4-Gfp* returned 35-40 million 80 base pair reads. Sequencing from E13.5 *Inha x Oct4-Gfp* returned 65-85 million 100 base pair reads. The *Inhba* and *Inha* datasets were analysed independently of each other. RNA sequencing data were processed and analysed by Monash University Bioinformatics Platform. Sequencing reads were aligned to the Ensembl mouse reference genome GRCm38 (Ensembl release 84) and analysis was performed using the RNAsik pipeline with STAR aligner (34). Differential gene analysis was performed on Degust V4.1.5 (David R. Powell, Monash Bioinformatics Platform), using *Limma-Voom* (35, 36). Heatmaps were generated using ClustVis (37). RNA-Seq data are available *via* accession number GSE201520.

*Inhba x Oct4-Gfp* analysis: Following principal component analysis of the samples, two samples were excluded as outliers: one sample from the E13.5 *Inhba* KO somatic cell group, and one sample from the E13.5 *Inhba* WT gonocyte group. Further scrutiny of these samples led us to conclude these may have been contaminated or swapped, and their exclusion was supported following consultation with a bioinformatician (Monash Bioinformatics Platform).

The detection limit was determined by calculating the median of the entire array of counts per million (cpm) values for the datasets. For the entire dataset (germ and somatic cells), the detection limit was determined as 2.2 cpm, while the detection limit for the germ cell only dataset was calculated at 2.391 cpm. Values greater than these were determined as being detectable. Analysis of these data confirmed the purity of the germ and somatic cell populations through absence or presence of *Ddx4* (germ cells), and *Sox9* and *Nr5a1* (somatic cells) (**Supplementary Figure 1A**). Absence of *Inhba* in knockout animals was confirmed in the somatic cell population with a 4-fold increase measured from E13.5 to E15.5 (**Supplementary Figure 1B**), consistent with previously published data (10, 12).

Differentially expressed genes were identified as having a false discovery rate (FDR) <0.05, a LogFC>0.585 and <-0.585 (i.e. a 1.5-fold change up or down, respectively), and two or more samples across genotypes being greater than the detection limit of 2.391cpm. There were 44 DEGs identified in the E15.5 *Inhba* KO gonocyte dataset. None were identified in the E13.5 *Inhba* KO dataset, therefore further analysis was performed to generate a list of transcripts that are altered in E13.5 gonocytes, described below. The data was processed in Degust using *Limma-Voom*, and the p-value was calculated within the software using the trimmed mean of M-values (TMM) normalised voom-transformed expression levels. Differentially expressed genes at E13.5 were identified using a LogFC> 0.585 and <-0.585, p-value <0.01 and restriction to at least two samples across wildtype and knockout animals being greater than the detection limit of 2.391 cpm. This approach enabled less abundant transcripts to be considered, and it resulted in the identification of 46 DEGs.

*Inha x Oct4-Gfp* analysis: Two wildtype and knockout littermate pairs were analysed using batch correction. Mitochondrial genes were filtered out as they were highly variable and genes with a minimum of 2 cpm in at least 2 samples included. Differentially expressed genes were identified by FDR<0.05 and LogFC>0.585. This led to the identification of 45 DEGs. Germ cell purity was assessed by the presence of *Ddx4* and absence of *Sox9* and *Nr5a1* (**Supplementary Figure 1C**). *Inha* genotypes were confirmed in the somatic cell population by qRT-PCR (**Supplementary Figure 1D**).

Gene lists obtained after analysis (**Supplementary Tables 1**–**3**) were submitted to the PANTHER classification system (38, 39) to identify molecular functions, biological processes, and protein classes of the DEGs. A Venn diagram was created following input of DEG lists to JVenn (40).

### Statistical Analysis

All statistical analyses were performed using GraphPad Prism Software (San Diego, CA, USA). Normal distribution of control and treatment groups was determined using a Shapiro-Wilk or D’Agostino and Pearson normality test. qRT-PCR and flow cytometric data from whole gonad cultures were analysed using an unpaired Student’s t-test for normally distributed data, or a Mann-Whitney test for data that was not normally distributed. For statistical analysis of the isolated cell culture experiments, a paired t-test or a Wilcoxon matched-pairs signed rank test was performed. Data was determined as significantly different when the p-value was less than 0.05.

## Results

### Germ Cells Express the Signalling Machinery to Respond to Activin A

Levels of transcripts encoding activin and Nodal ligands, signalling machinery and inhibitors were obtained from RNA-Seq analysis of germ and somatic cell populations collected from wildtype *Inhba* E13.5 and E15.5 testes (**Figure 1B**). These data reveal the complexity and dynamic nature of signalling potential of these selected components of the TGFβ superfamily within the testis during this window of development that is crucial to testis and embryo masculinization.

*Inhba* and *Inhbb*, encoding activin A and B subunits, respectively, were detected in somatic cells at both ages (**Figure 1B**), while *Inhbc* was below the detection limit in all samples (data not shown). *Inhba* increased 4-fold from E13.5 to E15.5 (73.8 ± 27.1 to 295.7 ± 69.7 cpm), and *Inhbb* levels were relatively constant (93.3 ± 6.7 cpm and 81.0 ± 10.9 cpm). At E13.5, *Nodal* was measured in germ cells, but not somatic cells, and it decreased to undetectable levels at E15.5 (**Figure 1B**). The levels of *Inhba*, *Inhbb* and *Nodal* were consistent with previous reports (12, 41). Transcripts encoding the Type 2 receptors for activin A, activin B and Nodal, *Acvr2a* and *Acvr2b*, were present in both somatic and germ cells at both ages highlighting the potential for each of these to respond, however *Acvr2a* was present at higher levels in both ages and cell types (**Figure 1B**). *Acvr1b*, encoding the type 1 receptor for activin A, activin B and Nodal, was present in both cell types at E13.5 and E15.5, while the transcript encoding the Nodal and activin B receptor, *Acvr1c*, was present only at low levels in E13.5 somatic cells (8.0 ± 2.3 cpm), indicating that *Acvr1b*, and not *Acvr1c*, is the predominant receptor for Nodal actions in germ cells at E13.5. Nodal signalling additionally requires the co-receptor, Cripto, encoded by *Tdgf1*, also known to antagonise activin A (42); this transcript was detected in E13.5 germ cells only (18.0 ± 1.2 cpm). These results illustrate the potential for Nodal to specifically impact on the germline cells which are exiting their proliferative state. Transcripts encoding the intracellular signalling components required for activin/Nodal signalling, *Smad2* and *Smad4*, were present at both ages in somatic and germ cells, however Smad3 was predominantly detected in the somatic cell samples (**Figure 1B**).

Activin and Nodal inhibitors are also present during fetal testis development, and these would be expected to fine-tune the responsiveness of cells expressing their receptors (**Figure 1A**). *Inha*, encoding the inhibin α subunit which forms a potent activin A inhibitor when dimerised with an activin β subunit, was detected only in somatic cells at both E13.5 (60.8 ± 8.1 cpm) and E15.5 (85.9 ± 34.7 cpm). Follistatin (*Fst*) was detected at low levels (<7 cpm) in all samples (**Figure 1B**), consistent with previous studies demonstrating that *Fst* is only expressed in the fetal ovary compared with the testis (43, 44). The transcript encoding the decoy receptor Bambi (45) was expressed at both ages in somatic and germ cells, with consistently higher levels in somatic cell samples compared with those in germ cells (53.6 ± 2.3 and 37.3 ± 1.7 cpm in somatic cells, and 9.7 ± 1.6 and 9.6 ± 2.4 cpm in germ cells). Transcripts encoding the inhibitory Smad6 and Smad7 were predominantly expressed in the somatic cells, but were also measured in germ cells at both ages. Betaglycan, encoded by *Tgfbr3*, is a co-receptor for TGFβs which is required for TGFβ2 signalling, and it can inhibit activin A (46). It was highly expressed in somatic cells compared to germ cells at both ages (**Figure 1B**).

There are several Nodal antagonists which could dampen its capacity to compete with activin A. *Lefty1* was identified in germ cells at E13.5 (**Figure 1B**; 63.6 ± 8.1 cpm) and undetectable by E15.5, consistent with previous observations (12). *Lefty2* was expressed at higher levels in E13.5 germ cells (192.8 ± 49.9 cpm) and dropped to 4.3 ± 1.5 cpm by E15.5. *Lefty2* transcripts were also low in the somatic cells at both ages (**Figure 1B**). The *Cerberus* transcript, encoding another Nodal inhibitor, was below the detection limit in all samples (data not shown). The Nomo/Nicalin complex has been identified as a Nodal antagonist in zebrafish (47), however its roles in the mouse are not known. Transcripts for each component were present in the mouse fetal testis (*Nomo1* and *Ncln*) at E13.5 and E15.5 in both somatic and germ cells (**Figure 1B**), indicating these proteins may also reduce Nodal activity in the fetal testis.

### Transcriptional Changes in Gonocytes in the Absence of Activin A (*Inhba* Knockout)

**Figure 2:**
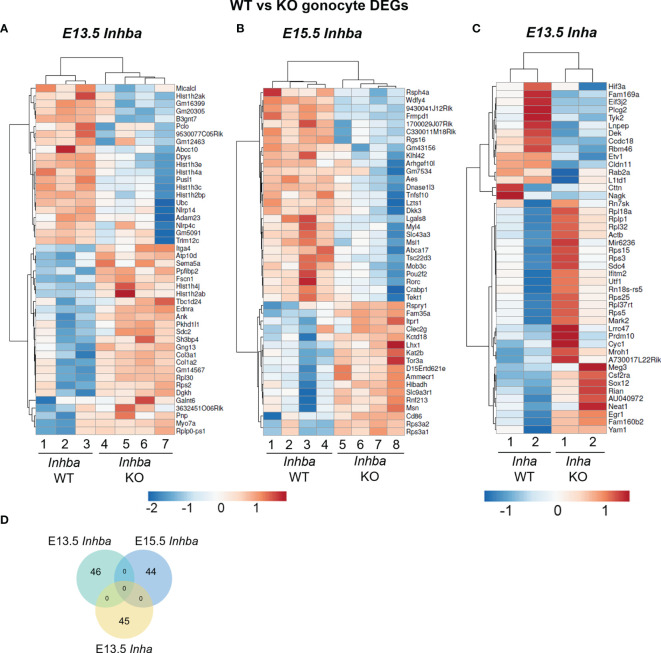
Heatmaps generated from differentially expressed genes (DEGs) following RNA-Seq analysis. **(A)** 15 downregulated and 30 upregulated DEGs in E13.5 *Inha* knockout (KO) gonocytes compared with wildtype (WT) littermates (n=2 per genotype). DEG criteria: FDR<0.05, LogFC>0.585, <-0.585. **(B)** 21 downregulated and 25 upregulated DEGs in E13.5 *Inhba* KO (n=3) compared with WT (n=4) gonocytes. DEG criteria: LogFC>0.585, p-value<0.01. **(C)** 27 downregulated and 17 upregulated DEGs in E15.5 *Inhba* KO compared with WT gonocytes (n=4 per genotype). DEG criteria: FDR<0.05, LogFC>0.585, <-0.585. **(D)** Venn diagram demonstrating lack of overlap of DEGs from the E13.5 and E15.5 *Inhba* KO, and E13.5 *Inha* KO gonocytes.

In germ cells lacking activin A (*Inhba* KO), there were 46 and 44 differentially expressed genes (DEGs) at E13.5 and E15.5, respectively (**Figures 2A, B**; **Supplementary Tables 1**, **2**). At E13.5, there were no DEGs by FDR (<0.05), therefore we utilised p-value (<0.01) and LogFC (>0.585 and <-0.585) to assess any differences between genotypes (**Figure 2A**). There were 21 downregulated, and 25 upregulated DEGs, which were primarily associated with binding and catalytic functions, and cellular processes. The top protein class was identified as being metabolite interconversion enzymes, which convert one small molecule to another (PANTHER, **Table 3**), however, the function of these genes in the testis are unknown. Interestingly, an association between *Galnt6* and piRNAs has been identified in an oral squamous cell carcinoma mouse model (48).

**Table 3:** PANTHER analysis of RNA-Seq DEGs.

PANTHER analysis	*Inhba* E13.5	*Inhba* E15.5	*Inha* E13.5
**Molecular function**	**Binding (GO:0005488)**	**Binding (GO:0005488)**	**Binding (GO:0005488)**
	Hist1h2ak	Dkk3	Rplp1
	Gng13	Pou2f2	Dek
	Fscn1	Mob3c	Tyk2
	Ubc	Lhx1	Cttn
	Sema5a	Slc9a3r1	Hif3a
	Tbc1d24	Rorc	Rps5
	Itga4	Msn	Etv1
	Myo7a	Crabp1	Lnpep
	**Catalytic activity (GO:0003824)**	Itpr1	Sox12
	Atp10d	**Catalytic activity (GO:0003824)**	L1td1
	Pusl1	Hibadh	Egr1
	Pnp	Mob3c	Rbm46
	Tbc1d24	Abca17	**Catalytic activity (GO:0003824)**
	Dgkh	Rspry1	Rplp1
	Dppys	Kat2b	Tyk2
	Myo7a		Plcg2
	Abcc10		Mark2
			Lnpep
			Nagk
**Biological processes**	**Cellular Process (GO:0009987)**	**Cellular Process (GO:0009987)**	**Cellular Process (GO:0009987)**
	Atp10d	Lzts1	Rplp1
	Sdc2	Dkk3	Dek
	Pusl1	Rsph4a	Tyk2
	Gng13	Arhgef10l	Ifitm2
	Fscn1	Pou2f2	Rps15
	Ubc	Mob3c	Cttn
	Sema5a	Lhx1	Plcg2
	Pclo	Rspry1	Hif3a
	Ppfibp2	Kat2b	Mark2
	Dgkh	Slc93r1	Rps5
	Itga4	Rorc	Etv1
	Dpys	Msn	Sdc4
	Myo7a	Cd86	Lrrc47
	Abcc10	Tekt1	Cldn11
	Rps2	Dnas1l3	Lnpep
		**Biological Regulation (GO:0065007)**	Sox12
		Lzts1	L1td1
		Dkk3	Egr1
		Arhgef10l	
		Pou2f2	
		Mob3c	
		Lhx1	
		Kat2b	
		Rorc	
		Msn	
		Cd86	
**Protein Class**	**Metabolite interconversion enzyme**	**Cytoskeletal protein**	**Translational protein**
	Pusl1	Rsph4a	Rplp1
	Pnp	Msn	Eif3j2
	Dgkh	Myl4	Rpl32
	B3gnt7	Tekt1	Rps15
	Dpys	**Gene-specific transcriptional regulator**	Rpl18a
	Galnt6	Pou2f2	Rps5
		Lhx1	Rps3
		Rorc	Lrrc47
		**Protein binding activity modulator**	Rps25
		Arhgef10l	**Gene-specific transcriptional regulator**
		Mob3c	Hif3a
		Rgs16	Etv1
		**Transporter**	Sox12
		Abca17	Egr1
		Itpr1	Prdm10
		Slc43a3	

At E15.5, there were 27 downregulated, and 17 upregulated DEGs (**Figure 2B**), however there was no overlap in DEGs between E13.5 and E15.5. These transcripts were similarly associated with binding and catalytic activity (PANTHER, **Table 3**). The top biological processes were cellular processes, and biological regulation, with the top protein classes identified as cytoskeletal proteins, gene-specific transcriptional regulators, protein binding activity modulators, and transporters (PANTHER, **Table 3**). Within the DEGs, Musashi-1 (*Msi1*), an RNA-binding protein in the Musashi family of proteins which function in translational regulation, was identified as lower in KO germ cells compared with WT counterparts. Its essential role in governance of postnatal transitions of murine spermatogenesis has been established, and it was previously shown to be expressed in gonocytes (49). There was no overlap in DEGs between E13.5 and E15.5, indicating age-specific responses of germ cells occurred in the absence of activin A.

A recent study identified that activin A promotes a less differentiated transcript profile in the human germ cell-like cell line, TCam-2 (30). To determine if germline differentiation was similarly altered in *Inhba* KO mice, we examined early and differentiation-associated germ cell transcripts in the *Inhba* WT and KO RNA-Seq dataset. Early germ cell transcripts *Nodal*, *Tdgf1*, *Kit*, *Lefty1*, *Lefty2*, and *Nanog* were all downregulated between E13.5 and E15.5 in both WT and KO samples, while differentiation markers *Nanos2* and *Dnmt3l* were upregulated (**Supplementary Figure 2**). We also observed higher expression of piRNA pathway transcripts such as *Piwil1, Piwil2* and *Piwil4, Dnmt3a, Dnmt3l, Tdrd1, Tdrd9, Mael and Mov10l1* at E15.5 relative to E13.5 in WT and KO samples. This is consistent with the activation of the piRNA pathway and *de novo* methylation from around E14.5-E15.5 in quiescent germ cells (50); the higher level of piRNA pathway transcripts encoding components such as in our dataset is consistent with the normal progression of developmental events associated with this phenomenon (**Supplementary Figure 2**). Of these transcripts, *Mov10l1* was decreased (p<0.05, Mann-Whitney test) in the KO germ cells at E15.5, however this was not determined to be differentially expressed in the RNA-Seq dataset by FDR and fold change, as presented in **Figure 2B**. Transcripts associated with pluripotency and differentiation showed no differences in isolated germ cells from *Inhba* KO testes compared with WT counterparts at either E13.5 or E15.5. The germ cell-specific transcripts, *Ddx4* and *Pou5f1* (*Oct4*), were both detected at relatively high levels in germ cells, with *Ddx4* increasing 1.6-fold from E13.5 to E15.5 in WT cells.

While *Inhba* KO germ cells appear to differentiate normally based on classical germ cell markers, a subset of genes was altered, indicating that loss of activin A modulates some aspects of early male germline transcription. However, the significance of the outcomes remains to be determined.

### The E13.5 Germ Cell Transcriptome Is Altered in the High Activin A Environment of the *Inha* Knockout Testis

The elevation of activin A levels linked with pre-eclampsia in human pregnancy can occur in the second and third trimesters when male germ cells are mainly quiescent. The inhibin α subunit encoded by *Inha*, forms a dimer with an INHBA subunit to form the inhibin A protein, a potent inhibitor of activin A. In *Inha* KO mice, activin A bioactivity is elevated due to the combined absence of inhibitory inhibin proteins, and to the greater availability of INHBA subunits for dimerization to form activin proteins. In wildtype mice, *Inhba* is detectable from E11.5, with its levels increasing until just after birth (10, 51). As the phenotype of the E13.5 testis appears normal but is significantly different by E15.5 (data not shown), we examined the germ cell transcriptome in *Inha* KO compared to WT littermates, prior to gross morphology changes. RNA-Seq analysis of germ cells isolated from two independent wildtype and knockout littermate pairs identified 45 DEGs (**Figure 2C** and **Supplementary Table 3**; FDR<0.05, LogFC>0.585, <-0.585). Thirty upregulated transcripts included ribosome structural components such as *Rps15, Rps25, Rps5, Rplp1* and *Rps3*. These transcripts are also associated with RNA binding. Pathway analysis revealed that the top molecular functions of the 45 DEGs were binding and catalytic activity, with cellular processes the top associated biological process. *Inha* KO DEG were associated with translational proteins (primarily the ribosomal structural component transcripts), and gene-specific transcriptional regulators, which included *Sox12*, *Egr1*, *Etv1* (upregulated), and *Prdm10* (downregulated) (**Table 3**). Interestingly, there were no reciprocal DEGs between *Inha* E13.5 germ cells and the E13.5 or E15.5 *Inhba* germ cells (**Figure 2D**). Collectively, these results demonstrate that gonocytes which develop in an environment of altered activin bioactivity are different from their wildtype counterparts, leading us to investigate whether this effect is direct or indirect.

### Germ Cells Can Respond Directly to Activin A

**Figure 3:**
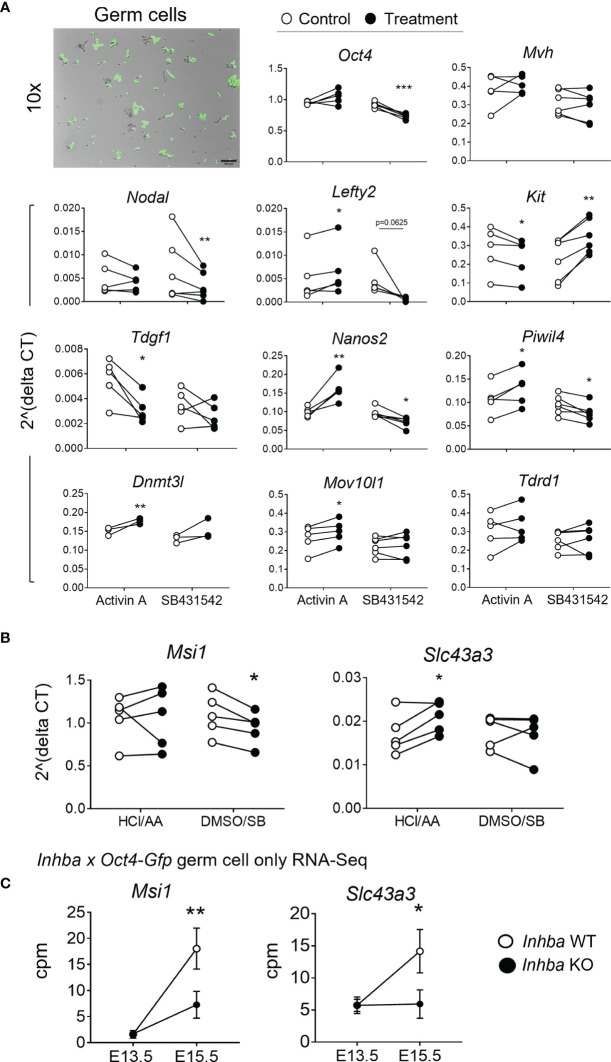
Germ cells can respond directly to activin A modulation. **(A)** E13.5 germ cells isolated *via* FACS were cultured with 5 ng/mL activin A or 10 µM SB431542 for 24 hours. Early germ cell and differentiation-associated transcripts were measured by qRT-PCR and normalised to *Canx* or the mean of *Canx* and *Mapk* housekeepers. Data was analysed using the 2^deltaCT method, and each individual experiment is graphed. Following the Shapiro-Wilk normality test, significance was determined by a paired t-test or Wilcoxon test and indicated by asterisks (*p < 0.05, **p < 0.01, ***p < 0.001). Top left-hand image represents brightfield and fluorescent overlay of Oct4-positive germ cells after 24 hours in culture. Germ cells were lightly adherent, accounting for the overlap shift. Scale bar represents 100 µm. **(B)** Transcript levels of Musashi-1 (*Msi1*) and *Slc43a3*, as described above. **(C)** Transcript levels of *Msi1* and *Slc43a3* in the *Inhba* x *Oct4-Gfp* E13.5 and E15.5 WT and KO germ cell RNA-Seq dataset (counts per million; cpm), presented as mean ± SD.

RNA-Seq revealed differences in male germ cell mRNA profiles in mice with altered activin A bioavailability (**Figure 2**). To test whether activin A can directly affect germ cells, gonocytes isolated from E13.5 testes were cultured for 24 hours in 5 ng/mL activin A or 10 µM SB431542, and appropriate vehicle controls. After 24 hours in culture, germ cells retained *Oct4-*eGFP expression, as observed by fluorescence microscopy (**Figure 3A**). Transcripts encoding markers of germ cell differentiation were measured in isolated E13.5 gonocytes and first compared with levels in cells cultured for 24 hours in control conditions (**Supplementary Figure 3**). After 24 hours in culture, the early germ cell marker *Kit* had declined to 85% of E13.5 levels, and *Nodal* was at 10% of E13.5 levels. The differentiation marker *Nanos2* was moderately increased (1.8-fold), while *Dnmt3l, Piwil4, Tdrd1* and *Mov10l1* were all higher after 24 hours in culture compared with E13.5 levels (16-, 11-, 6- and 3-fold, respectively). Interestingly, germ cell markers *Oct4* and *Mvh* increased over time (**Supplementary Figure 3**). The decrease in *Nodal* and increase in differentiation markers suggests that E13.5 germ cells can autonomously differentiate outside of the somatic environment.

*Oct4* and *Mvh* transcripts were unaffected by activin A exposure, however SB431542 resulted in a significant decrease in *Oct4* (0.8-fold). *Nodal* and *Lefty2* levels were also unaffected by activin A exposure, however both were lower in SB431542-treated cells, consistent with previous reports (12). *Kit* was significantly lower following activin A exposure (0.85-fold), and significantly higher following SB431542 (1.75-fold), and *Tdgf1*, encoding the Nodal co-receptor, was significantly reduced by activin A (**Figure 3A**). The mRNA encoding the Nodal inhibitor Lefty2 is a known activin A-responsive gene, demonstrated in mouse embryonic stem cells and P19 embryonic carcinoma cells (52, 53), and in human TCam-2 cells (30). This responsiveness was also demonstrated here in isolated gonocytes, with a 1.61-fold increase in *Lefty2* following activin A exposure (**Figure 3A**).

In addition, treatment of E13.5 gonocytes with activin A resulted in a more differentiated transcript profile, with significant elevation of *Nanos2*, *Piwil4*, *Dnmt3l* and *Mov10l1*. Further, SB431542 decreased *Nanos2*, consistent with whole gonad culture, and *Piwil4*, while *Kit* increased These results demonstrate that gonocytes respond directly to activin A and the inhibition of its pathway in culture (**Figure 3A**).

Two transcripts, Musashi-1 (*Msi1*) and Solute carrier family 43 member 3 (*Slc43a3*), identified as DEGs in the RNA-Seq data from E15.5 activin A knockout mouse testes (**Figure 2B**), were investigated in these samples. Following exposure to SB431542, *Msi1* was significantly decreased to 0.86-fold of control levels in E13.5 gonocytes after 24 hours, but it was not affected by activin A (**Figure 3B**). In *Inhba* WT germ cells, *Msi1* normally increases 10-fold between E13.5 and E15.5. This was reduced to a 4-fold increase between E13.5 and E15.5 in *Inhba* KO germ cells, resulting in a significant difference in expression levels between wildtype and knockout germ cells at E15.5 (60% decrease, **Figure 3C**). In the isolated somatic cells of *Inhba WT* and *Inhba* KO testes, examined using RNA-seq, the level of *Msi1* recorded was greater than in germ cells (>20 cpm; data not shown) (9) but was not different between genotypes. Thus, *Msi1* appears to be a germ cell-specific activin A target gene, a conclusion supported by the results in the E13.5 isolated germ cell cultures in which *Msi1* was significantly decreased following activin/Nodal/TGFβ inhibition, and that it was significantly reduced in germ cells of *Inhba* KO animals at E15.5.

Slc43a3, originally identified as an equilibrative nucleobase transporter, has also been identified as influencing fatty acid flux (54, 55) but its function in the testis is unknown. *Slc43a3* was lower in the E15.5 *Inhba* KO germ cells compared to WT (**Figures 2B**, **3C**), and was significantly higher in activin A-treated gonocytes (1.26-fold of controls) (**Figure 3B**) suggesting that it is upregulated by activin A directly in germ cells. While *Slc43a3* was not altered following SB431542 exposure; this may be due to a difference between the chronic absence of activin A in the *Inhba* KO mouse and acute inhibition in these cultures *via* SB431542. It is also important to consider that the germ cells may be developmentally different, or that *Slc43a3* transcript may be relatively stable and therefore not reduced within the 24-hour window examined in the isolated E13.5 germ cells.

### Dose-Dependent Response of Activin A Somatic Target Genes in Whole Testis Culture

**Figure 4:**
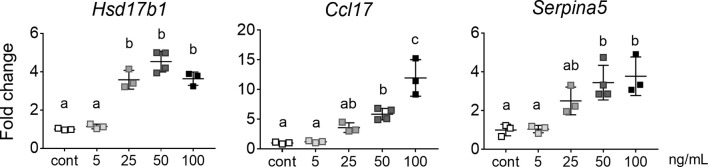
Dose-dependent response of activin A target genes. qRT-PCR analysis of transcripts of E13.5 whole gonads exposed to 5 to 100 ng/mL activin A for 48 hours in culture and compared with control (n=3 or 4 per group). Data were normalised to the *Canx* housekeeper and expressed as fold change compared to control (no activin A). Data are presented as mean ± SD, and significance was determined by one-way ANOVA following the Shapiro-Wilk normality test. Significant differences are indicated by different letters.

After determining that gonocytes can directly respond to activin A and SB431542 through altered gene expression, we cultured whole testes to assess the outcome of altered signalling on germ cells within their somatic niche. We first performed a dose-response, to determine the optimal concentration of activin A. E13.5 testes were cultured with 5, 25, 50 or 100 ng/mL activin A for 48 hours and compared with control samples cultured in the vehicle. Levels of known activin A-induced somatic cell transcripts, *Hsd17b1*, *Ccl17* and *Serpina5* (9), were monitored to determine the optimal dose at which responses were evident. *Hsd17b1* was significantly higher in testes exposed to 25, 50 and 100 ng/mL, while *Ccl17* and *Serpina5* were significantly higher in testes exposed to 50 and 100 ng/mL of activin A, when compared with vehicle controls (**Figure 4**). Because all three transcripts were increased following exposure to at least 50 ng/mL activin A, this concentration was chosen for subsequent experiments.

### Acute Effects of Activin A and SB431542 on Somatic Cells

**Figure 5:**
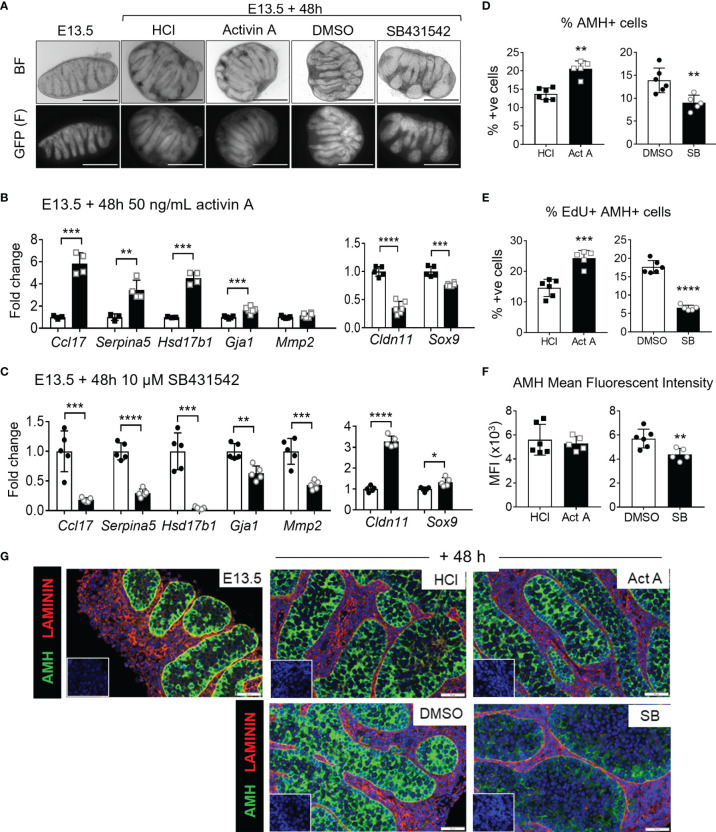
Acute activin A and SB431542 effects on fetal testicular somatic cells. E13.5 testes were cultured for 48 hours on membranes. **(A)** Representative brightfield (top panel) and fluorescent (GFP, bottom panel) images of E13.5 testis and E13.5 testes cultured 48 h with 50 ng/mL activin A, 10 µM SB431542, or vehicle controls (HCl and DMSO respectively). **(B)** Somatic cell transcripts of individual testes (n=3-5 per group) cultured for 48 hrs with 50 ng/mL activin A (black bars) compared with control (white bars). **(C)** Somatic cell transcripts of individual testes (n=3-5 per group) cultured for 48 hrs with 10 µM SB431542 (black bars) compared with control (white bars). All transcripts were measured by qRT-PCR and data normalised to *Canx* housekeeper. **(D–F)** Flow cytometry analysis of dissociated E13.5 testes cultured with 50 ng/mL activin A or 10 µM SB431542 for 48 hrs compared with controls. Proportion of **(D)** AMH-positive (Sertoli) cells, **(E)** EdU-positive AMH-positive (proliferating Sertoli) cells, and **(F)** mean fluorescent intensity (MFI) of AMH-positive population. **(G)** Representative images of AMH (green) and Laminin (red) immunofluorescence staining of 48hr-cultured E13.5 testes. Sections were counterstained with DAPI (blue) for nuclear detection and scale bar is 50 µm. All graphical data are presented as mean ± SD and significant differences determined by a Student’s t-test or Mann-Whitney test following the Shapiro-Wilk normality test and indicated by asterisk (*p < 0.05, **p < 0.01, ***p < 0.001, ****p < 0.0001).

E13.5 testes were cultured with 50 ng/mL activin A or with the activin/Nodal/TGFβ inhibitor, SB431542, which blocks ligand access to the Type 1 receptors, ALK4, ALK7 and ALK5 (56). Testes were photographed immediately after collection at E13.5 and after 48 hours of culture. Testis cords were easily observed in the E13.5 testes, and after 48 hours of culture in either vehicle, the cords appeared elongated, contained GFP-positive germ cells, and were grossly of the shape normally observed *in vivo* at E15.5 (**Figure 5A**). In contrast, after 48 hours in culture the effectiveness of inhibitor treatment was evident based on the appearance of cords that were fatter and appear stunted, compared with the DMSO controls (**Figure 5A**), previously demonstrated by Miles and colleagues in cultures beginning at E12.5 (12). Cords in testes cultured with activin A were grossly similar to control testes but appeared to be slightly thinner. Activin A target gene transcripts were measured by qRT-PCR. *Ccl17*, *Serpina5*, *Hsd17b1* and *Gja1* (encoding gap-junction protein Connexin 43, expressed in Sertoli cells) were significantly higher than in corresponding control samples following activin A exposure (5.8-, 3.5-, 4.5- and 1.7-fold, respectively; **Figure 5B**), and significantly lower in SB431542-treated testes (0.18-, 0.3-, 0.04- and 0.63-fold of control) (**Figure 5C**), confirming the efficacy of these treatments and demonstrating a dose-dependency of these transcript levels as previously reported *in vivo* (9). *Cldn11*, also encoding a component of Sertoli cell tight junctions, decreased in post-pubertal rat Sertoli cell *in vitro* cultures following activin A exposure (22). The finding that *Cldn11* was significantly lower in activin A-treated fetal testes (0.36-fold), and significantly increased in SB431542-treated testes (3.3-fold) (**Figures 5B, C**) indicates that the responsiveness of these genes to activin bioactivity is likely to be conserved through the Sertoli cell lifespan.

Matrix metalloproteinases are involved in tissue remodelling and have been detected in the fetal testis (57, 58). Exposure of the human gonocyte-like seminoma cell line, TCam-2, to activin A increased both MMP2 transcript and protein levels (59). Therefore, *Mmp2* was also assessed as a potential activin A target in the mouse fetal testis. Activin A exposure did not alter *Mmp2* transcript in fetal mouse testes, however SB431542 significantly decreased 0.43-fold of controls (**Figures 5B, C**). *Mmp2* may not be solely upregulated by activin A, as its decrease following SB431542 exposure could be due to the inhibition of TGFβs or Nodal. Alternatively, *Mmp2* synthesis could have already been at the highest level normally reached by activin A stimulation by the levels present at E13.5. Opposing regulation of *Ccl17, Serpina5*, *Hsd17b3, Gja1* and *Cldn11* by activin A and SB431542 demonstrates the effectiveness of each in culture, while extending our knowledge of how transcripts encoding extracellular matrix components are regulated in the fetal gonad.

To assess Sertoli cells, *Sox9* transcription was measured following whole gonad culture with activin A or SB431542. Interestingly, *Sox9* transcript was significantly lower in activin A-treated gonads (0.76-fold) and significantly higher (1.33-fold) in SB431542-treated gonads (**Figures 5B, C**). This was consistent with our RNA-sequencing analysis of fetal somatic cells from *Inhba* KO mice (data not shown) which collectively suggests that *Sox9* transcription or turnover may be modulated by activin A.

Testes in fetal mice lacking activin A have a reduced proportion of proliferative Sertoli cells (10, 19), and E12.5 testes exposed to SB431542 for 72 hours exhibited a five-fold decrease in Sertoli cell proliferation (12). To assess the effects of activin A and SB431542 on cell proliferation in cultured whole testes, Edu-incorporation followed by flow cytometry was employed. Fetal Sertoli cells, detected by AMH immunostaining, comprised 14% of the total cell population after 48 hours in culture with vehicle controls (HCl, DMSO); testes exposed to activin A had a significantly higher proportion of fetal Sertoli cells, with a 1.5-fold increase to 21% of AMH-positive cells. Conversely, SB431542 exposure significantly reduced the proportion of Sertoli cells to 9% (0.65-fold of DMSO levels) (**Figure 5D**). Consistent with this, we observed a significant increase in the proportion of EdU-positive Sertoli cells following activin A exposure (1.7-fold), demonstrating that activin A increased Sertoli cell proliferation, and a decrease following SB431542 exposure (0.37-fold), demonstrating decreased Sertoli cell proliferation (**Figure 5E**). In addition, the mean fluorescent intensity (MFI) of AMH in AMH-positive cells was also measured as an indication of relative protein levels; Flow cytometric analysis revealed that SB431542 significantly reduced the AMH MFI (**Figure 5F**), and this was confirmed in sections of SB431542-treated testes analysed using immunofluorescence staining (**Figure 5G**).

### Testis Culture Supports Germ Cell Development

**Figure 6:**
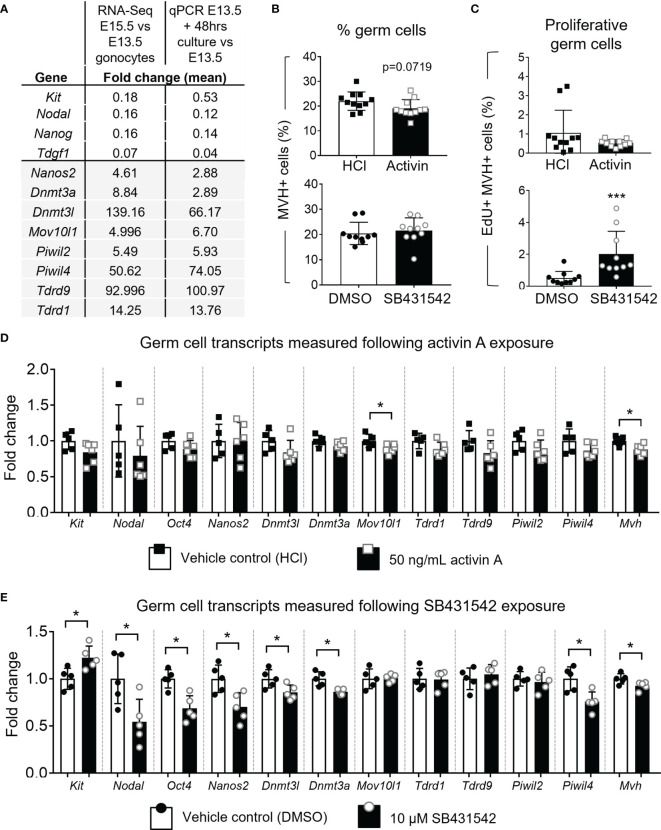
Effect of acute activin A and SB431542 exposure on germ cells. E13.5 testes were cultured for 48 hours on membranes. **(A)** Table presents the fold change of transcript levels. The second column presents fold change of E15.5 compared to E13.5 germ cell transcripts measured by RNA-Sequencing (counts per million (CPM); n=3-4 animals per age). The third column represents the transcript fold-changes of E13.5 testes cultured under normal conditions for 48 hours compared with E13.5 testes measured by qRT-PCR (data was normalised to *Canx*; n=5 per group). **(B, C)** Flow cytometry analysis of E13.5 testes cultured with 50 ng/mL activin A or 10 µM SB431542 for 48 hours compared with respective vehicle controls (HCl and DMSO). **(B)** Proportion of germ cells (MVH+ SOX9-) and **(C)** EdU-positive proliferating germ cells following culture with activin A or SB431542 (black columns) compared with controls (white columns). For activin A and controls, n=5 per group, for SB431542 and controls, n=5 and n=4 respectively. **(D, E)** qRT-PCR analysis of early germ cell and differentiation-associated markers in individual E13.5 testes cultured with **(D)** 50 ng/mL activin A (black bars; n=6) or HCl control (white bars; n=5) or **(E)** 10 µM SB431542 (black bars) or DMSO control (white bars; n=5 per group). Transcripts measured by qRT-PCR were normalised to *Canx* housekeeper and fold change compared to control group shown. All graphical data are presented as mean ± SD and significant differences determined by a Student’s t-test or Mann-Whitney test following the Shapiro-Wilk or D’Agostino and Pearson normality tests and indicated by asterisk (*p < 0.05, ***p < 0.001).

The *Oct4-Gfp* transgene allowed visualisation of germ cells by fluorescent microscopy after culture. Based on GFP localisation, germ cells appeared restricted to the cords (**Figure 5A**). Levels of germ cell transcripts, assessed by qRT-PCR, were compared between E13.5 whole testes and testes cultured for 48 hours in vehicle. These were also examined against the RNA-seq data of wildtype E13.5 and E15.5 gonocyte populations isolated from *Inhba* x *Oct4-Gfp* mice. Early germ cell transcripts *Kit, Nodal, Nanog* and *Tdgf1* were lower in testes after 48 hours in culture compared with E13.5 testes, and the differentiation markers *Nanos2, Dnmt3a, Dnmt3l, Mov10l1, Piwil2, Piwil4, Tdrd9* and *Tdrd1*, normally upregulated by E15.5, were all increased after 48 hours in culture. These findings were consistent the changes measured by RNA-Seq (**Figure 6A**) and demonstrate the suitability of the culture system for investigating effects on germ cell development.

### A Small Proportion of Gonocytes Escape Mitotic Arrest Following SB431542-Exposure

Treatment of E12.5 testes with 10 µM SB431542 for 72 hours previously resulted in an increased proportion of germ cells escaping mitotic arrest, with a 4-fold increase (3% to 14%) in germ cells incorporating EdU (12). This indicates that blocking ALK4/5/7 signalling has a robust effect on mitotic arrest. To assess the window of vulnerability of germ cells to this disruption, and to assess whether the proportion of germ cells in this sub-population was sustained, we investigated whether E13.5 testes were similarly susceptible to SB431542 treatment, an age when most germ cells have already entered mitotic arrest. In parallel, we sought to determine whether exposure to exogenous activin A would influence germ cell numbers or proliferation. For these studies, EdU-incorporation and flow cytometry were employed detect proliferating MVH-positive (germ) cells after 48 hours in culture. Germ cells comprised approximately 20% of the total cell population in the cultured testes. There were no significant differences in this value between treatment and control samples after 48 hours in culture, but there was a trend to fewer germ cells following activin A treatment (0.86-fold, p=0.0719) (**Figure 6B**). The proportion of Edu^+^ germ cells in the SB431542 treatment group was increased (2% Edu^+^, compared with controls, 0.5% Edu^+^) (**Figure 6C**). This was statistically significant, and indicates that a small proportion of germ cells in E13.5 testes retain the capacity to escape mitotic arrest. Moreover, together the observations that SB431542 diverts a greater proportion of the germ cell population from mitotic arrest at E12.5 compared to E13.5, indicate that there is a window at around E12.5 during which inhibiting AKL4/5/7 can divert germ cells from their normal entry into mitotic arrest.

### Activin/Nodal/TGFβ Inhibition in E13.5 Mouse Testes Promoted a Less-Differentiated Germ Cell Phenotype

To further examine the relevance of this pathway to fetal germ cell differentiation in these whole fetal testis cultures, key markers were measured by qRT-PCR. Early germ cell marker transcripts *Nodal*, *Kit* and *Oct4* were not different following activin A treatment, and amongst key transcripts normally upregulated between E13.5 and E15.5 (*Nanos2*, *Dnmt3l*, *Dnmt3a*, *Mov10l1*, *Tdrd1*, *Tdrd9*, *Piwil2* and *Piwil4*), only *Mov10l1* was affected and was 11% lower than in the control sample. However, the germ cell marker *Mvh* was reduced by 12% following activin A treatment (**Figure 6D**).

**Figure 7:**
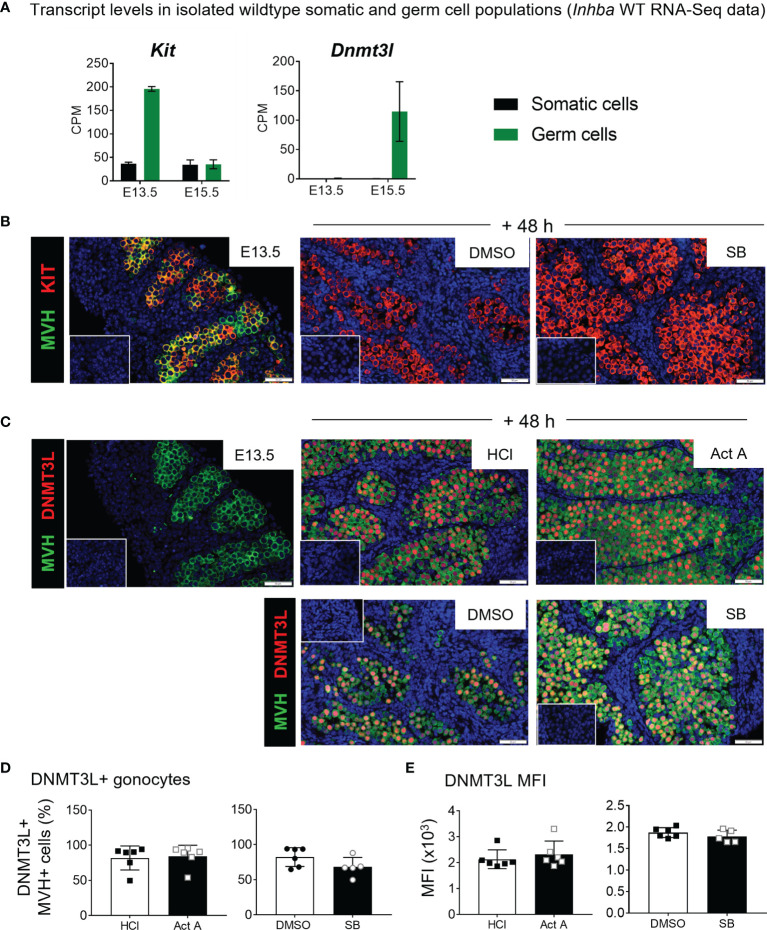
KIT and DNMT3L expression in E13.5 testis cultures. **(A)** E13.5 and E15.5 transcript levels of *Kit* and *Dnmt3l* from FACS-sorted *Inhba* WT somatic and germ cell RNA-Seq data, expressed in counts per million (CPM). **(B, C)** Immunofluorescence staining of E13.5 and cultured testes. **(B)** Detection of KIT (red) and MVH (green) marking germ cell cytoplasm in E13.5 testis and E13.5 testes cultured for 48 hours with 10 µM SB431542 or DMSO control. **(C)** Detection of DNMT3L (red) and MVH (green) in E13.5 testis and those cultured 48 hours with 50 ng/mL activin A or 10 µM SB431542 and respective vehicle controls. DAPI staining in blue marks nuclei. Scale bars are 50 µm, insets represent controls lacking primary antibody. **(D, E)** Flow cytometry measuring **(D)** proportion of DNMT3L-positive MVH-positive germ cells and **(E)** mean fluorescent intensity (MFI) of DNMT3L-positive population following 48 hr culture with activin A or SB431542. All graphical data are presented as mean ± SD and significant differences determined by a Student’s t-test or Mann-Whitney test following the Shapiro-Wilk or D’Agostino and Pearson normality tests.

E13.5 testes exposed to 10 µM SB431542 exhibited a less-differentiated transcript profile. *Nodal* is highly expressed at E13.5 in germ cells and decreases to <20% by E15.5 (**Figure 1B**). After 48 hours of culture with SB431542, *Nodal* was downregulated to 54% of the control level (**Figure 6E**). Nodal upregulates its own expression (32, 41), and because SB431542 blocks Nodal signalling through ALK4/5/7 inhibition, this downregulation of *Nodal* was expected, and consistent with findings from Miles and colleagues (12). The early germ cell marker *Kit* was significantly higher following SB431542 exposure (1.22-fold, compared with controls; **Figure 6E**). While *Kit* is also expressed in somatic cells, the *Inhba* KO RNA-Seq data shows that at E13.5, *Kit* is predominantly expressed in germ cells (196 ± 5 cpm vs 36 ± 4 cpm in somatic cells; **Figure 7A**) suggesting that the increase in *Kit* is most likely due to an effect of activin/Nodal/TGFβ inhibition on germ cells.

In SB431542-treated testes, the gonocyte differentiation marker *Nanos2* was reduced to 70% of controls, and several PIWI/piRNA pathway components, which normally increase by E15.5 when germ cells are quiescent, were also reduced. The *de novo* DNA methyltransferases *Dnmt3a* and *Dnmt3l* were both reduced by 14% to 86% of control levels in SB431542-treated gonads compared to controls. Similarly, *Piwil4* was reduced to 76% of controls, but there was no change in *Mov10l1*, *Tdrd1*, *Tdrd9* or *Piwil2* levels. The germ cell markers *Oct4* and *Mvh* were reduced to 69% and 93% of control levels following SB431542 exposure, however there was no change in germ cell numbers (**Figure 6E**). Collectively, these changes indicate a modest transcriptional response of these genes to activin/Nodal/TGFβ inhibition.

Because the early germ cell marker *Kit* was upregulated, and the differentiation marker *Dnmt3l* was downregulated following SB431542 exposure, immunofluorescence staining for these two markers was performed on E13.5 testes, and on the activin A and SB431542 treatment samples. KIT was co-localised with MVH in E13.5 germ cells, corresponding with transcript data, but was not detectable in SB431542-treated testes (**Figure 7B**), despite transcript up-regulation. *Dnmt3l* values in germ and somatic cells at E13.5 are below 1 cpm and increase in germ cells to 115 ± 50 cpm at E15.5 (**Figure 7A**). By immunofluorescence, DNMT3L was not detectable in any MVH-positive germ cells at E13.5 but was detected in the nucleus of germ cells after 48 hours of culture in every treatment group, consistent with its normal upregulation by E15.5 (**Figure 7C**). There were no obvious differences between activin A- or SB431542-treated testes compared with their respective controls. DNMT3L appeared to be heterogeneously distributed, with bright and dim staining present in individual germ cell nuclei (**Figure 7C**), however flow cytometry revealed no difference in DNMT3L-positve germ cells (**Figure 7D**) or its MFI between treatment groups (**Figure 7E**). Further scrutiny of the data did not reveal any distinct “bright” or “dim” populations, nor differences in their distribution across treatment groups.

These data suggest that inhibition of activin/Nodal/TGFβ activity in E13.5 testes cultured for 48 hours results in a less-differentiated germ cell transcript profile. Considering that a small subpopulation of germ cells escaped mitotic arrest in SB-treated gonads (**Figure 6C**), it is possible that the changes observed in the transcript profiles may reflect only the small population of germ cells that have not yet entered quiescence.

### Delineating Direct and Indirect Effects of Activin A and SB431542 on Gonocytes

**Figure 8:**
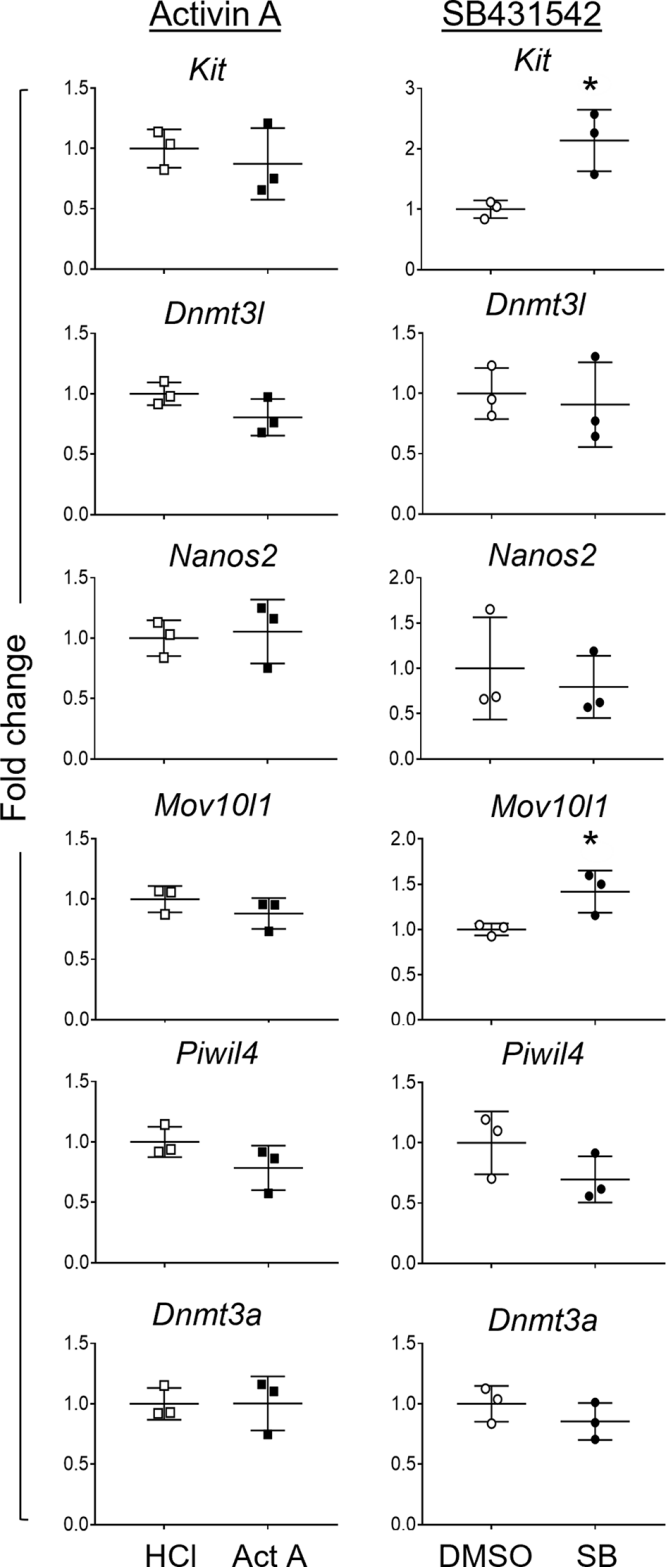
Germ cell transcripts in gonocytes isolated after 48-hour whole testis culture. Germ cells were isolated *via* FACS from E13.5 testes cultured for 48 hours with 50 ng/mL activin A (Act A, black squares) or 10 µM SB431542 (SB, black circles) and their controls (white symbols). Transcripts of germ cell genes were measured by qRT-PCR. Treatment groups are presented as fold change compared to control (n=3 individual testes per group). Data were normalised to the *Canx* housekeeper gene, and presented as mean ± SD. Significance was determined by a Mann-Whitney test or Student's t-test following the Shapiro-Wilk normality test, and indicated by asterisk (*p < 0.05).

After documenting the impact on fetal germ cells of chronic activin A disruptions in transgenic mouse models and demonstrating that isolated germ cells in culture can respond directly to activin A, we wanted to extend our knowledge of how exogenous activin A and SB431542 exposures each affect the germ cells within the intact testis environment. Whole E13.5 testes were dissociated after 48h culture with activin A or SB431542, and the gonocytes isolated by FACS for transcript analysis. Known activin A target genes were analysed in the isolated somatic cells to confirm the effectiveness of activin A and SB431542 treatments in the cultures. These results were consistent with the previous whole gonad cultures (**Supplementary Figure 4**). Transcript analysis of isolated gonocytes after whole testis culture with activin A revealed no changes in the early germ cell (*Kit*), or differentiation (*Dnmt3l, Nanos2, Mov10l1, Piwil4* or *Dnmt3a*) markers. SB431542 exposure resulted in significantly increased *Kit* levels (2-fold), consistent with whole testis analysis and isolated germ cell cultures (**Figure 8**). Interestingly, SB431542-exposure did not result in decreased levels of the differentiation marker transcripts *Dnmt3l*, *Nanos2*, *Piwil4* or *Dnmt3a*. Unexpectedly, *Mov10l1* was significantly increased (1.4-fold; **Figure 8**). These data suggest that, while gonocytes can directly respond to perturbed activin A and TGFβ signalling, the effects are minimized whilst they reside in an intact somatic environment.

## Discussion

The outcomes of this study have demonstrated that germ cells respond directly and indirectly to conditions with which levels of activin A, or its signalling pathway are altered. These findings highlight the value of understanding the contribution of TGFβ superfamily crosstalk to the complex processes required for normal testis development, as the inhibition of receptors shared by activin A, Nodal and TGFβ had a robust impact. The effects of acute pathway inhibition on germ cells was more prominent in intact testes: they displayed a delayed differentiation profile and a smaller proportion of germ cells entered mitotic arrest. Although analyses of intact E13.5 testes cultured for 48 hours with activin A identified minimal effects on germ cells, those germ cells isolated from mice with chronically altered activin A levels have altered transcriptomes at both E13.5 and E15.5. Analysis of cells isolated from *Inhba* mutant mice at two different ages provides evidence of age-specific readouts of activin A signaling, as there was no overlap in DEGs between the ages. A general comparison of what were expected to be equivalent samples (i.e. duplicates at each genotype and age) show variations that would be expected for transcripts that are undergoing dynamic regulation at each of these time points. While culture of isolated germ cells demonstrated their cell-intrinsic capacity to directly respond, with exogenous activin A promoting advanced differentiation transcript profiles, we did not observe reciprocal gene expression changes in the two models. This may reflect signalling interactions between activin A and other pathways.

Murine male germ cells enter mitotic arrest starting from E13.5, and the vast majority are quiescent by E15.5 (3, 26). In the present study, a small but significantly higher population of germ cells (2% of population) were identified as mitotic (in S-phase) in E13.5 whole testes cultured with SB431542 for 48 hours compared with controls. A similar analysis of E12.5 testes cultured with SB431542 for 72 hours reported that approximately 20% of germ cells escaped mitotic arrest, an outcome not observed using the TGFβ-specific inhibitor Alk5i; this result indicated that entry into quiescence was selectively disrupted by activin and/or Nodal signalling in these cultures (12). Thus, the findings in this study are consistent with previous reports, and identify the potential for TGFβ signalling disruptions to alter the maturation pace of fetal male germ cells, including by allowing a small proportion of germ cells to delay mitotic arrest. This may be relevant to human pathologies that arise from disruptions to the differentiation of just a small number of cells. It is well documented that altered activin A signalling disrupts normal testicular somatic cell development, with the Sertoli cells the main target of activin A actions (9, 10, 18, 19). Because spermatogenic development is reliant on the somatic niche, germ cells are susceptible to local environmental changes that could include changes to hormones, growth factors, and extracellular matrix composition which influence somatic cell functions. In humans, arrested or disrupted differentiation of fetal germ cells is deemed to underpin the emergence of the GCNIS cells which can progress to form testicular germ cell tumours in young men (60). Therefore, minor disruptions to TGFβ signalling could lead to significant consequences in adulthood that may be more impactful in species such as humans which have a long pre-pubertal period.

The combined inhibition of several ligands using SB431542 resulted in a stronger phenotypic change in both culture systems. It is therefore important to consider the combined actions of TGFβ superfamily ligands on testis development and their potential for functional redundancy. TGFβs have a role in regulating germ cell proliferation in the testis. Exogenous TGFβ1 and TGFβ2 decrease the number of gonocytes and increase the number of apoptotic germ cells in fetal rat testis cultures (61). In mouse, 24-hour cultures of E13.5 testes with TGFβ2 decreased gonocyte numbers, and blocking TGFβ signalling in germ cells *in vivo* increased the proportion of proliferative germ cells (11). Treatment of E11.5 and E12.5 XX gonads with a combination of FGF9, TGFβ1, activin A and activin B led to a greater induction of male characteristics than did exposure to a single ligand (62).

Germ cells isolated from E13.5 testes autonomously continue to develop in culture in the absence of a somatic environment. This was previously documented, as E13.5 male germ cells cultured up to 6 days upregulated *de novo* DNA methylation, autonomously establishing genomic imprints (63). The capacity for isolated germ cells to develop in different culture conditions (collagen-coated inserts with 20% serum in the Iwahashi study, vs on plastic with 10% serum, used here) suggest that fetal germ cells harbour a robust cell-autonomous developmental program. In the present study, isolated gonocytes exposed to activin A decreased the early germ cell marker *Kit* and increased differentiation markers such as *Nanos2* and *Piwil4*. In contrast, SB431542 exposure increased *Kit*, and decreased *Nanos2* and *Piwil4*. These data are consistent with, and extend the findings by Wu and colleagues (64).

In both whole testes and isolated germ cells cultured with SB431542, *Kit* levels were increased, consistent with a delayed differentiation profile. In contrast, *Oct4*, a pluripotency marker, was lower in SB431542-treated testes and isolated germ cells. However, Nodal has been shown to promote *Oct4* transcription in a mouse spermatogonial cell line (65), and *Oct4* has been demonstrated to be a direct target of SMAD2 binding in mouse ES cells (52). Therefore, inhibition of activin/Nodal/TGFβ signalling may negatively regulate *Oct4* levels. While isolated germ cells retain their differentiation trajectory, they are sensitive to external signalling cues such as altered TGFβ superfamily signalling.

The importance of identifying targets of activin A signalling relates to the value of understanding how *in utero* environmental exposures may impact on adult fertility. Entry into quiescence signifies a key differentiation step of fetal germ cells and coincides with an increase in the differentiation marker *Nanos2* and of transcripts encoding PIWI/piRNA pathway components such as DNMT3L, DNMT3A and PIWIL4. The decreased levels of these transcripts in E13.5 testes exposed to SB431542 during an interval when they would normally be increasing indicates their differentiation is delayed. The PIWI/piRNA pathway plays an important role in the genomic methylation of retrotransposons during epigenetic reprogramming (5, 50). Mice lacking either PIWIL4 or DNMT3L are sterile, and to various degrees exhibit reduced methylation and increased levels of transposable elements (50, 66, 67), and DNMT3A methylates the maternally imprinted *H19* gene (68). *Mov10l1*, essential for the primary processing of piRNA precursors that have translocated to the cytoplasm (69), is decreased in *Inhba* KO E15.5 gonocytes and increased in activin A-treated gonocytes. Interestingly, *Mov10l1* was increased in germ cells isolated following whole testis culture with SB431542. Loss of primary piRNAs in *Mov10l1* mutant mice completely disrupts the PIWI/piRNA pathway, leading to de-repression of retrotransposons and increased levels of LINE1 and IAPs in postnatal germ cells (70). Similar to other mouse models with genetic modifications of the PIWI/piRNA pathway, the absence of *Mov10l1* causes male-specific sterility (50, 71–73). Because the consequences of PIWI/piRNA pathway disruption often severely affect fertility, it will be useful to determine if the functional consequences of aberrant activin A signalling include altered DNA methylation, increased levels of retrotransposons or reduced levels of piRNAs in germ cells.

*Musashi-1* (*Msi1*) encodes an RNA-binding protein, first characterised in *Drosophila* as a regulator of germ cell stemness (74) and shown to impact on germline development in the postnatal testis in mice. MSI1 is present in the cytoplasm of gonocytes and spermatogonia, and in the nucleus of the more differentiated pachytene spermatocytes. Its overexpression impairs spermatogenesis a finding linked to its role in nuclear delivery of an mRNA required for meiotic progression (49, 75). The present study identified *Msi1* reduction in E15.5 germ cells of *Inhba* mutant mice (lacking activin A), and also in E13.5 isolated germ cells exposed to SB431542, providing the first evidence that *Msi1* may be a novel target of activin/TGFβ superfamily signalling.

The somatic cell environment is ultimately essential for fetal germ cell development and therefore crucial to consider when investigating the effect of signalling pathways on testis growth. Anti-Mullerian hormone (AMH), produced by fetal Sertoli cells from E12.5 until puberty (76), is essential for Mullerian duct regression. In the present study, AMH protein levels measured by immunofluorescence on sections, was markedly reduced in SB431542-treated testes. This is in accordance with the report that exposure of human first trimester testes to SB431542 for two weeks in a hanging drop culture system abolishes the AMH signal in cells and reduces its secretion into the media (77). Interestingly, MMP2 is also essential for Mullerian duct regression, and mice lacking AMH have decreased *Mmp2* expression in Mullerian ducts (78). Mouse testes also exhibited reduced *Mmp2* levels following SB431542 treatment, which may be a consequence of reduced AMH levels. This result highlights the challenges inherent in delineating indirect versus direct signalling pathway outcomes. Gonads of AMH-deficient mice have been examined, but only up to E12.5; testis morphology appeared normal (79), however they did not assess later development *in utero*, when the testis cords are expanding and elongating. AMH is phylogenetically conserved, and the ortholog is present in species that lack Mullerian ducts, such as fish. In medaka fish, AMH is essential for regulating germ cell proliferation; loss-of-function mutations result in excessive proliferation and premature meiosis in male fish (80). It will be of interest to determine the roles of AMH on both somatic and germ cells within the fetal testis.

The integration of cellular development in the fetal testis provides the foundation for ongoing spermatogenesis throughout adulthood. This study has shown that gonocytes can respond directly to activin A and its inhibition. Chronic absence or elevation of activin A can alter the gonocyte transcriptome, and combined activin, Nodal and TGFβ inhibition leads to a less-differentiated phenotype. Importantly, it appears that the somatic cell environment can dominate, and potentially attenuate, gonocyte responsiveness to altered TGFβ superfamily pathway signalling. The use of several complimentary approaches will be required to fully discern how fetal germ cells develop normally in response to somatic cues and to understand the impact of inappropriate cues arising from maternal exposures or genetic factors. Studies such as this one capitalise on the general similarities in the developmental chronology of mouse and human testis growth to learn about germ cell development. The identification of activin A target genes, in addition to others potentially affected by TGFβ superfamily signalling disruptions, provides the opportunity to unearth how germ cells respond to signalling cues and potential outcomes within the complex cellular milieu of the fetal testis. Such information can ultimately identify processes that are of relevance to human pathologies.

## Data Availability Statement

The datasets presented in this study can be found in online repositories. The names of the repository/repositories and accession number(s) can be found below: NCBI, GSE201520.

## Ethics Statement

The animal study was reviewed and approved by Monash University Animal Ethics Committee (Monash Medical Centre).

## Author Contributions

SM, KL, and PSW designed the experiments. SM performed experiments. PAFW assisted with RNA-Seq material collection. SM performed primary analysis, with KL and PSW performing critical review of results. SM and KL and PSW wrote the manuscript. All authors read and approved the final version of the manuscript.

## Funding

This work was supported by the National Health and Medical Research Council (NHMRC) of Australia grants (Ideas grant ID1181516 to KL, and project grant ID1081987 to KL and PSW). SM was supported by an Australian Government Research Training Stipend. This work also received support from the Victorian State Government Operational Infrastructure Scheme.

## Conflict of Interest

The authors declare that the research was conducted in the absence of any commercial or financial relationships that could be construed as a potential conflict of interest.

## Publisher’s Note

All claims expressed in this article are solely those of the authors and do not necessarily represent those of their affiliated organizations, or those of the publisher, the editors and the reviewers. Any product that may be evaluated in this article, or claim that may be made by its manufacturer, is not guaranteed or endorsed by the publisher.
