# Supplementary material for: The Impact of Activin A on Fetal Gonocytes: Chronic Versus Acute Exposure Outcomes
Source: Front Endocrinol (Lausanne). 2022 May 31;13:896747. doi: 10.3389/fendo.2022.896747 (PMC9205402; doi:10.3389/fendo.2022.896747)
Supplement: Supplementary file 1 [file DataSheet_1.pdf]

Supplementary Material

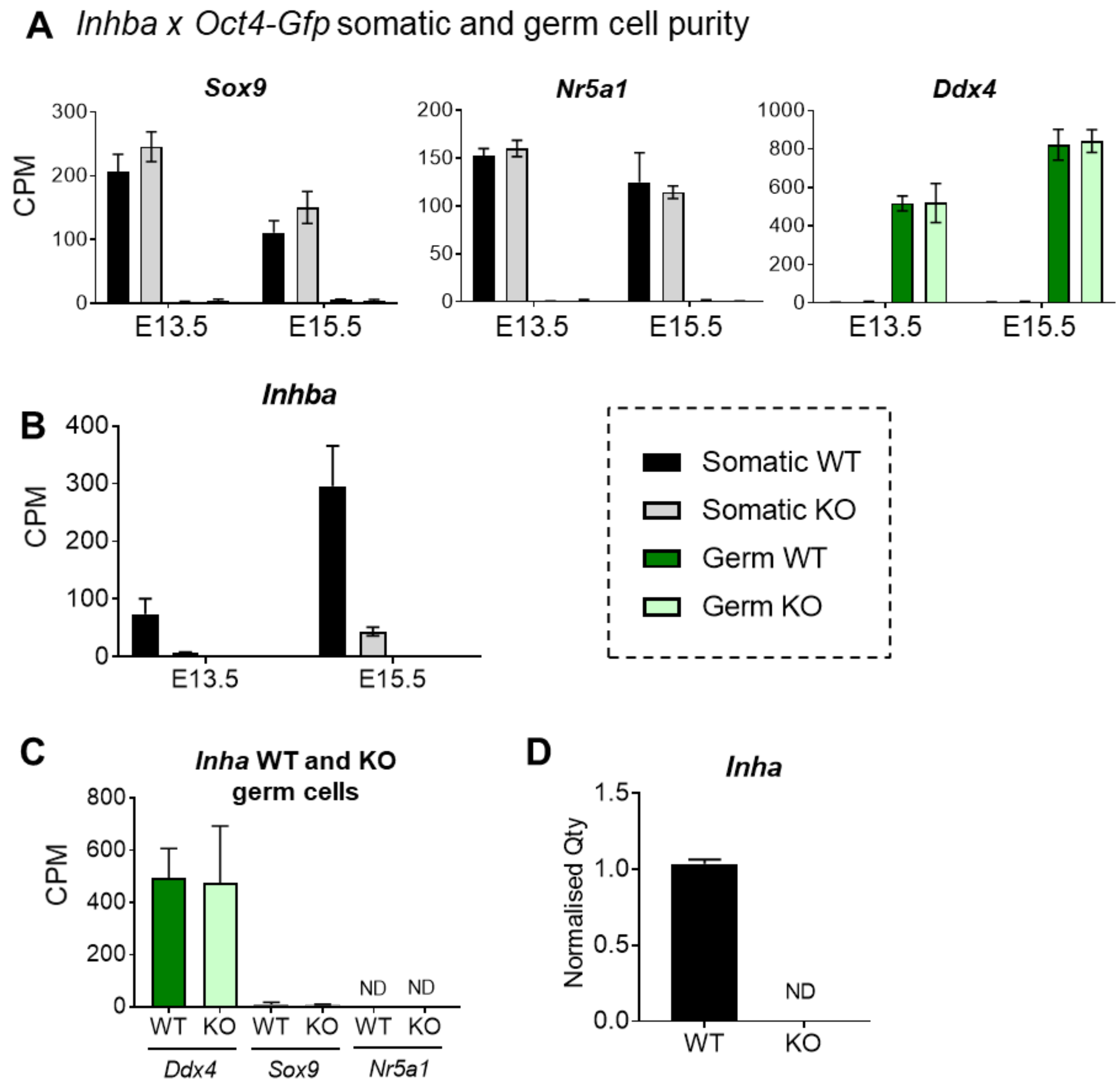

Supplementary Figure 1. Purity of germ and somatic cell populations, and confirmation of sample genotypes. (A) Expression of the Sertoli cell and somatic cell-specific transcripts, *Sox9* and *Nr5a1* respectively, and germ cell-specific transcrtipt *Ddx4* were measured by RNA-Seq in FACS-sorted somatic and germ cells of E13.5 and E15.5 testes from *Inhba* WT and KO animals. (B) Expression of *Ddx4, Sox9* and *Nr5a1* in *Inha* WT and KO E13.5 FACS-isolated germ cells. (C) Expression of *Inhba*, encoding activin A, in the germ and somatic cell populations of *Inhba* WT and KO testes. Data is expressed as counts per million (CPM) and presented as mean ± SD (A-C). (D) *Inha* transcript expression, measured by qRT-PCR, in isolated somatic cells from *Inha* WT (n=2) and KO (n=2) testes. qPCR data were normalised to the geometric mean of the *Canx* and *Mapk1* housekeeper genes, and expressed as mean ± SD.

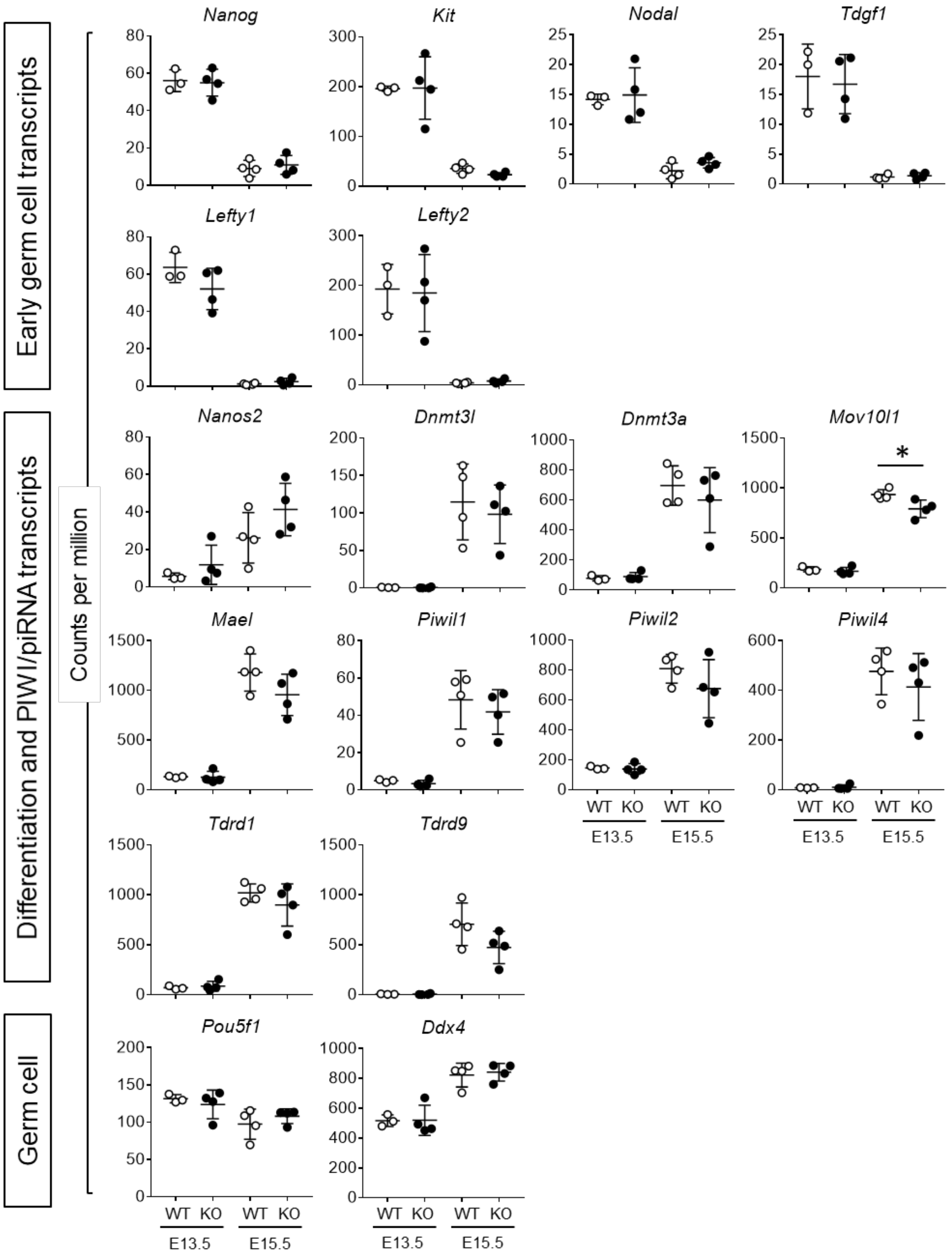

Supplementary Figure 2. Early and differentiation-associated germ cell transcripts in E13.5 and E15.5 *Inhba-Oct4Gfp* WT and KO germ cells. RNA-Sequencing of germ cells isolated from E13.5 and E15.5 *Inhba-Oct4Gfp* WT (open circles) and KO (black circles) testes. Counts per million of early germ cell transcripts, differentiation and PIWI/piRNA pathway genes were examined to assess normal development and changes between WT and KO germ cells. The germ cell markers *Mvh* and *Oct4* are also presented. Data are presented as mean ± SD. Significance was determined by Student's t-test or Mann-Whitney test following Shapiro-Wilk normality test. $^*p<0.05$.

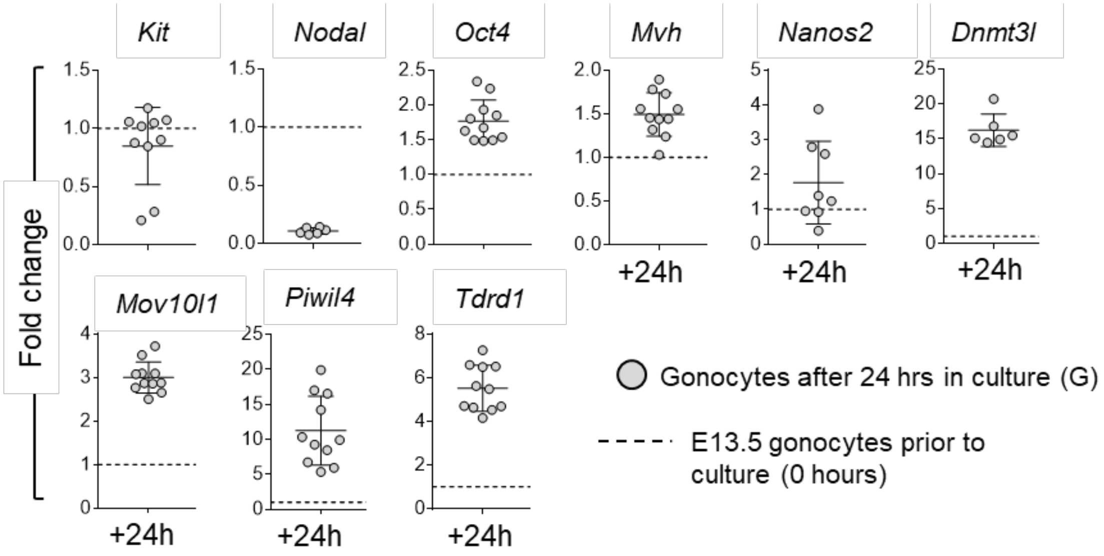

Supplementary Figure 3. Germ cell transcripts in isolated germ cell cultures over 24 hours. Transcript analysis by qRT-PCR of E13.5 gonocytes isolated by FACS and cultured for 24 hours in normal conditions. Fold change of each experiment (24 hours in culture compared with E13.5 gonocytes) is presented, with E13.5 gonocytes at 0 hours indicated by dotted line. Data are presented as mean ± SD.

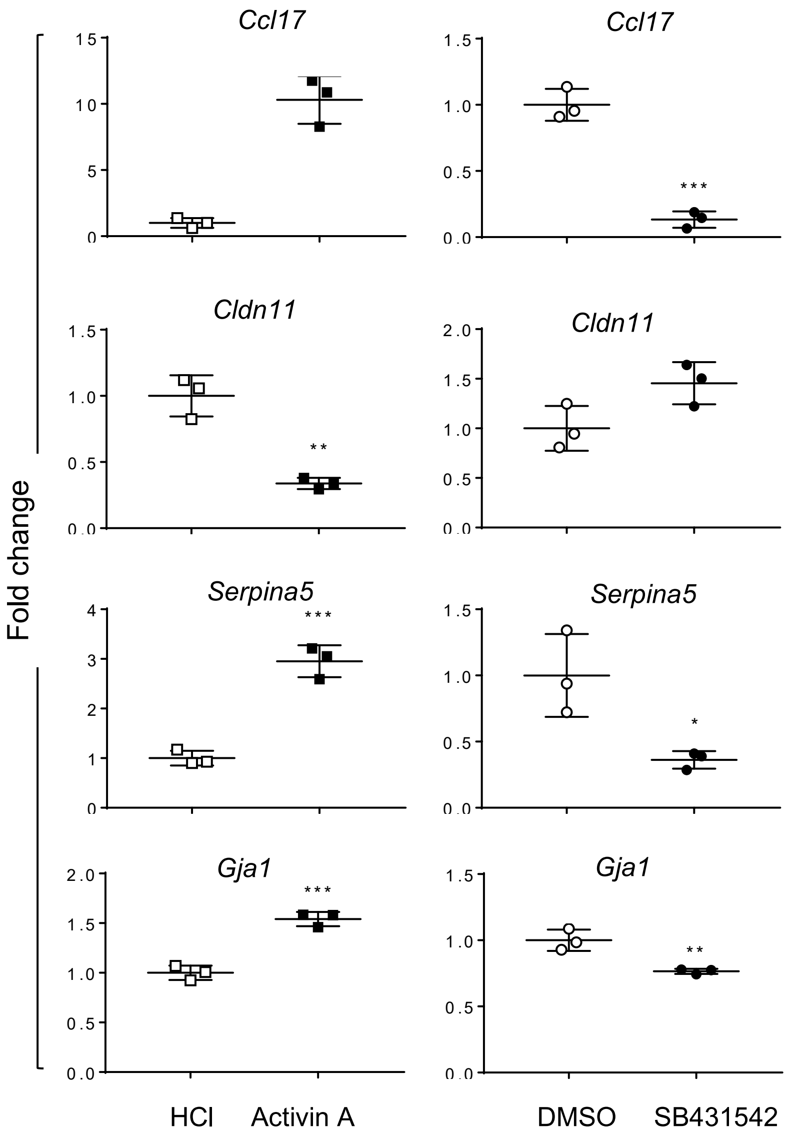

Supplementary Figure 4. Activin A target genes in somatic cells isolated from E13.5 testes cultured for 48h. Germ and somatic cells were isolated via FACS from E13.5 testes cultured for 48 hours with 50 ng/mL activin A (black squares) or 10 µM SB431542 (black circles) and their controls (white circles). Transcripts of somatic activin A target genes were measured by qRT-PCR to confirm the effect of activin A and SB431542 in culture. Treatment groups are presented as fold change compared to control (n=3 individual testes per group). Data were normalised to the *Canx* housekeeper gene, and presented as mean ± SD.
